# 3-hydroxy-L-kynurenamine is an immunomodulatory biogenic amine

**DOI:** 10.1038/s41467-021-24785-3

**Published:** 2021-07-21

**Authors:** Cristina C. Clement, Angelo D’Alessandro, Sangeetha Thangaswamy, Samantha Chalmers, Raquel Furtado, Sheila Spada, Giada Mondanelli, Federica Ianni, Sarah Gehrke, Marco Gargaro, Giorgia Manni, Luisa Carlota Lopez Cara, Peter Runge, Wanxia Li Tsai, Sinem Karaman, Jorge Arasa, Ruben Fernandez-Rodriguez, Amanda Beck, Antonio Macchiarulo, Massimo Gadina, Cornelia Halin, Francesca Fallarino, Mihaela Skobe, Marc Veldhoen, Simone Moretti, Silvia Formenti, Sandra Demaria, Rajesh K. Soni, Roberta Galarini, Roccaldo Sardella, Gregoire Lauvau, Chaim Putterman, Kari Alitalo, Ursula Grohmann, Laura Santambrogio

**Affiliations:** 1 Department of Radiation Oncology, Weill Cornell Medicine, New York, NY USA; 2 Department of Biochemistry and Molecular Genetics, University of Colorado Denver, Anschutz Medical Campus, Aurora, CO USA; 3 Division of Rheumatology and the Department of Microbiology and Immunology, Albert Einstein College of Medicine, New York, NY USA; 4 Department of Medicine and Surgery, University of Perugia, Perugia, Italy; 5 Department of Pharmaceutical Sciences, University of Perugia, Perugia, Italy; 6 Department of Pharmaceutical & Organic Chemistry, Faculty of Pharmacy, University of Granada, Granada, Spain; 7 Institute of Pharmaceutical Sciences, ETH Zurich, Zurich, Switzerland; 8 Translational Immunology Section, National Institute of Arthritis Musculoskeletal and Skin Diseases, Bethesda, MD USA; 9 Wihuri Research Institute and Translational Cancer Medicine Research Program, University of Helsinki, Helsinki, Finland; 10 Department of Oncological Sciences and Tisch Cancer Institute, Icahn School of Medicine at Mount Sinai, New York, NY USA; 11 Department of Pathology, Albert Einstein College of Medicine, New York, NY USA; 12 Instituto de Medicina Molecular João Lobo Antunes, Faculdade de Medicina da Universidade de Lisboa, Lisbon, Portugal; 13 Istituto Zooprofilattico Sperimentale dell’Umbria e delle Marche “Togo Rosati”, Perugia, Italy; 14 Sandra and Edward Meyer Cancer Center, New York, NY USA; 15 Proteomics and Macromolecular Crystallography Shared Resource, Herbert Irving Comprehensive Cancer Center, Columbia University Irving Medical Center, New York, NY USA; 16 Azrieli Faculty of Medicine, Bar-Ilan University, Zefat, Israel; 17 Research Institute, Galilee Medical Center, Nahariya, Israel; 18 Caryl and Israel Englander Institute for Precision Medicine of Weill Cornell Medicine, New York, NY USA

**Keywords:** Autoimmunity, Inflammation, Skin diseases

## Abstract

Tryptophan catabolism is a major metabolic pathway utilized by several professional and non-professional antigen presenting cells to maintain immunological tolerance. Here we report that 3-hydroxy-l-kynurenamine (3-HKA) is a biogenic amine produced via an alternative pathway of tryptophan metabolism. In vitro, 3-HKA has an anti-inflammatory profile by inhibiting the IFN-γ mediated STAT1/NF-κΒ pathway in both mouse and human dendritic cells (DCs) with a consequent decrease in the release of pro-inflammatory chemokines and cytokines, most notably TNF, IL-6, and IL12p70. 3-HKA has protective effects in an experimental mouse model of psoriasis by decreasing skin thickness, erythema, scaling and fissuring, reducing TNF, IL-1β, IFN-γ, and IL-17 production, and inhibiting generation of effector CD8^+^ T cells. Similarly, in a mouse model of nephrotoxic nephritis, besides reducing inflammatory cytokines, 3-HKA improves proteinuria and serum urea nitrogen, overall ameliorating immune-mediated glomerulonephritis and renal dysfunction. Overall, we propose that this biogenic amine is a crucial component of tryptophan-mediated immune tolerance.

## Abstract (web summary)

3-hydroxy-L-kynurenamine (3-HKA) is a metabolite deriving from a lateral pathway of tryptophan catabolism. Here the authors identify 3-HKA as a biogenic amine and show it has anti-inflammatory properties that can protect mice against psoriasis and nephrotoxic nephritis.

## Introduction

Indole derivatives are entwined in the most important processes of life. l-tryptophan (Trp), a typical indole compound, is an essential part of proteins and the precursor of a huge number of metabolites, mainly produced along the kynurenine pathway^1,2^. Although some Trp metabolites are well known for their activity in mammals as neurotransmitters (i.e., serotonin), hormone/chronobiotic (melatonin), neuroprotective agents (kynurenic acid), and neurotoxins (quinolinic acid), the functional meaning of several derivatives is still unknown. Indoleamine 2,3-dioxygenase 1 (IDO1) catalyzes the first, rate-limiting step of the kynurenine pathway and is highly expressed in antigen-presenting cells (APCs), such as dendritic cells (DCs), in inflammatory conditions dominated by interferon γ (IFN-γ). IDO1 has been considered an immune checkpoint mechanism capable of promoting peripheral immune tolerance^1–6^. On one hand, IDO1^+^ DCs deplete the microenvironment of Trp, an essential amino acid absolutely required for T-cell clonal expansion, thus inducing anergy. On the other, the Trp metabolites (i.e., kynurenines) produced downstream IDO1 can actively induce immune tolerance through additional mechanisms involving the development and expansion of regulatory T (Treg) cells and the concurrent dampening of inflammatory T helper 1 (Th1)- as well as Th17-mediated responses^1,3^. Therefore, in principle, the IDO1 potent immunoregulatory effects could be exploited in the control of autoimmune/chronic inflammatory diseases. However, the identification of the best immunoregulatory kynurenine with drug-like properties is still unclear.

Here we report the discovery of the previously undescribed biogenic amine 3-hydroxykynurenine (3-HKA), which is produced via a lateral branch of the IDO1 pathway in DCs, but also at high levels in lymphatic endothelial cells (LEC) and human tumor cell lines. 3-HKA inhibits the activation of pro-inflammatory STAT1 and NF-kB pathways in mouse and human DCs and has therapeutic effects in experimental models of psoriasis and nephrotoxic nephritis.

## Results and discussion

### 3-hydroxy-L-kynurenamine is a tryptophan metabolite

**Fig. 1:**
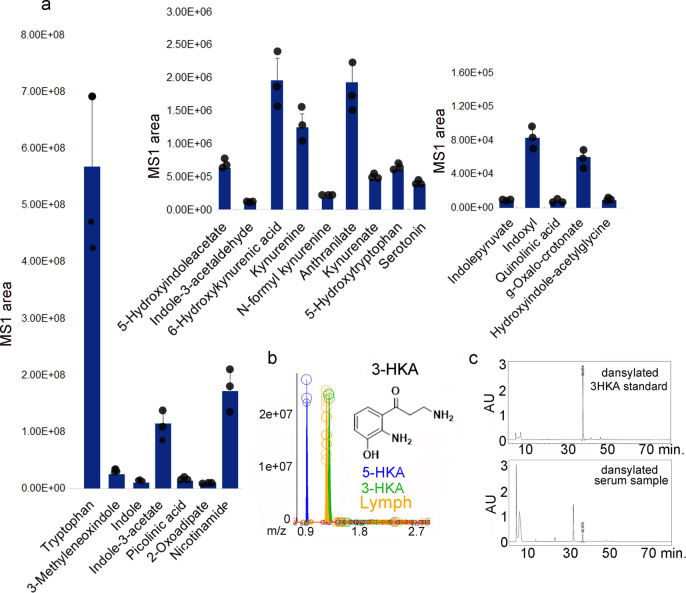
Lymphatic fluid contains several tryptophan metabolites. **a** Bar graphs reporting the MS1 area of tryptophan metabolites detected in the human lymph. Bar graphs represent MS1 average readings ± SD of *n* = 3 biological replicates. Source data are provided as a Source data file. **b** Standards used for identification/quantification of 3-hydroxykynurenamine (3-HKA) vs 5-hydroxy-l-kynurenamine by mass spectrometry. **c** 3-HKA was reacted with dansylchloride producing the di-dansylated 3-hydroxykynurenamine derivative (Supplementary Figs. 1 and 2). The stable derivatization product was quantitatively analyzed by HPLC-DAD.

**Fig. 2:**
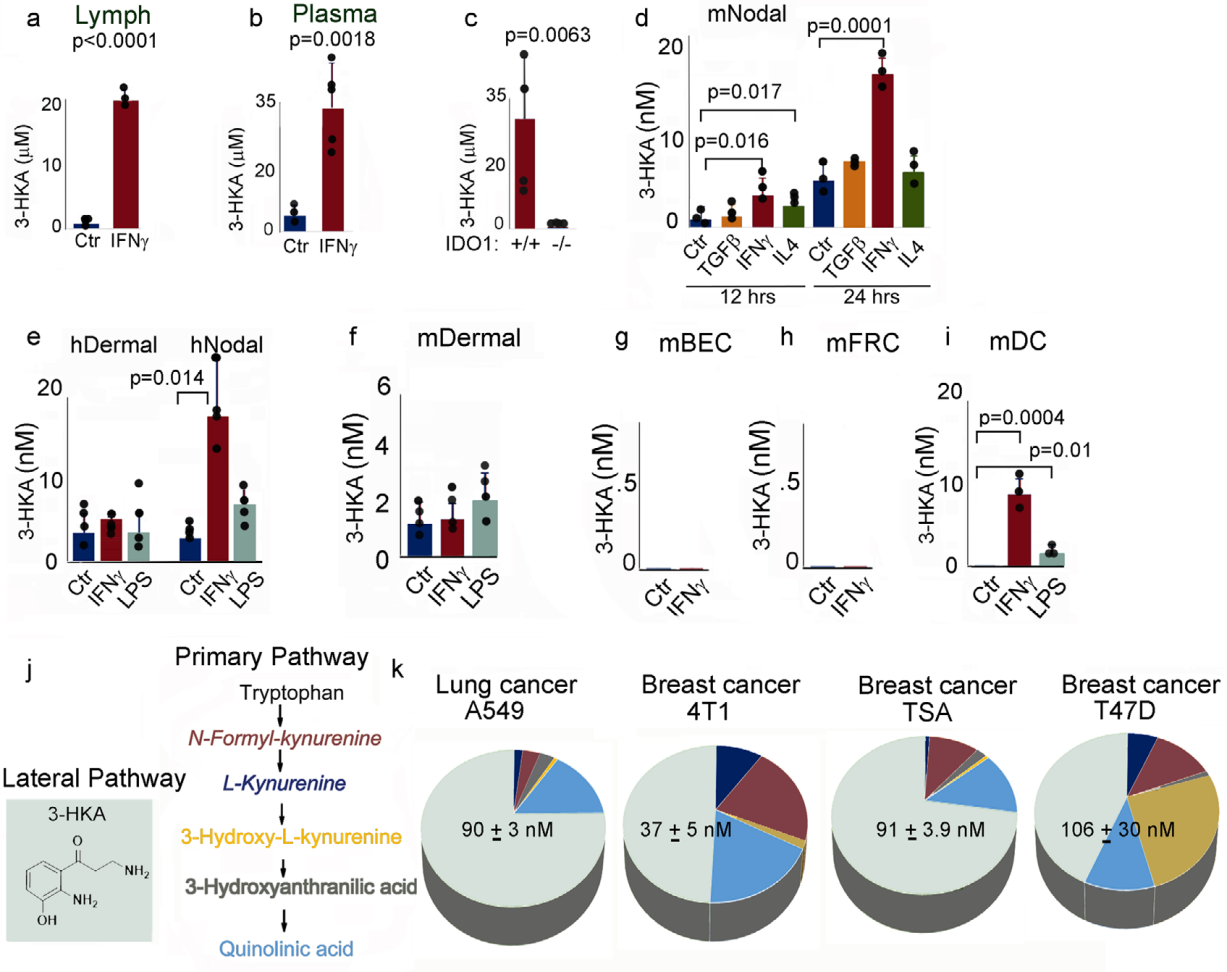
3-HKA is generated in vivo and in vitro following IFN-γ treatment. **a**, **b** Quantification of 3-HKA in lymph and plasma before and after i.p. injection of IFN-γ (5 μg). Lymph (*n* = 3 biologically independent samples) and blood samples (*n* = 3 and *n* = 5 biologically independent samples) were statistically analyzed using a two-tailed paired or unpaired student’s *t* test. **c** Quantification of 3-HKA in the plasma of C57BL/6J mice and *Ido*1 ko mice. Average ± SD of *n* = 4 biologically independent samples, statistically analyzed using a two-tailed paired student’s *t* test. **d** Nodal mouse LECs were cultured in serum-free media for 12 or 24 h with the indicated cytokines. 3-HKA is reported as average concentration ± SD of *n* = 3 biologically independent samples, statistically analyzed using a two-tailed paired student’s *t* test. **e** Nodal and dermal human LECs and mouse dermal LEC were cultured in serum-free media for 24 h with or without IFN-γ (100 ng/ml) or LPS (1 μg/ml). 3-HKA concentration is reported as average concentration ± SD of *n* = 4 biologically independent replicates statistically analyzed using a two-tailed paired student’s *t* test. **f** Dermal mouse LECs were cultured in serum-free media for 24 h with or without IFN-γ (100 ng/ml) or LPS (1 μg/ml). Data are reported as average 3-HKA concentration ± SD of *n* = 4 biologically independent replicates, statistically analyzed using a two-tailed paired Student’s *t* test. **g**, **h**, **i** Mouse blood endothelial cells (BEC) and fibroblast reticular cells (FRC) were cultured in serum-free media for 24 h with or without IFN-γ (100 ng/ml) and supernatant analyzed as in (**e**). **i** Mouse splenic CD11c-purified dendritic cells (DCs) were cultured in serum-free media for 24 h with or without IFN-γ (50 ng/ml) or LPS (1 μg/ml) and supernatant analyzed as in (**e**). Data are reported as average 3-HKA concentration ± SD of *n* = 3 biologically independent replicates, analyzed using a two-tailed paired Student’s *t* test. **j**, **k** Mouse 4T1 and TSA breast carcinoma and human A549 lung and T47D breast carcinoma cells were analyzed for the presence of Trp metabolites (percentage of each metabolite in the supernatant is reported color-coded for each line). 3-HKA concentration ± SD of *n* = 3 biologically independent replicates. Source data for (**a**–**k**) are provided as Source data file.

Untargeted metabolomic analysis of the lymphatic fluid identified the presence of several metabolites deriving from the IDO1, IDO2, and TDO-mediated Trp catabolism (Fig. 1a). Among those, metabolomic analysis detected the presence of a previously unreported amine, 3-hydroxy-L-kynurenamine (3-HKA) (Fig. 1b). To confirm the position of the hydroxyl group, we synthesized both the 3- and 5-HKA isomers to validate the results via UHPLC-MS (Fig. 1b). The two isomers were baseline separated under our analytical conditions and the lymph metabolite matched the 3-HKA standard with respect to its chromatographic retention time, with an accurate intact mass at a concentration of ~1 µM (Fig. 1b). To further validate the chemical nature of this metabolite, we utilized dansylation, a chemical procedure used to separate polyamines (Fig. 1c, Supplementary Figs. 1 and 2). Dansylated 3-HKA was prepared and used as an MS standard to detect and quantify endogenous dansylated 3-HKA present in biological fluids (Fig. 1c, and Supplementary Figs. 1 and 2). Since Trp catabolism is highly induced by IFN-γ, C57BL/6 mice were injected ip with 5 μg of mouse IFN-γ. After, 24 h, lymph was collected from the thoracic duct and blood by retro-orbital bleeding. In both biological fluids, micromolar 3-HKA amounts were detected at baseline condition and, in both cases, the concentration increased up to 40 μM following IFN-γ treatment (Fig. 2a, b). Importantly, no 3-HKA was observed in the plasma of IDO1-KO mice confirming that this enzyme is pivotal in the 3-HKA synthetic pathway (Fig. 2c).

**Fig. 3:**
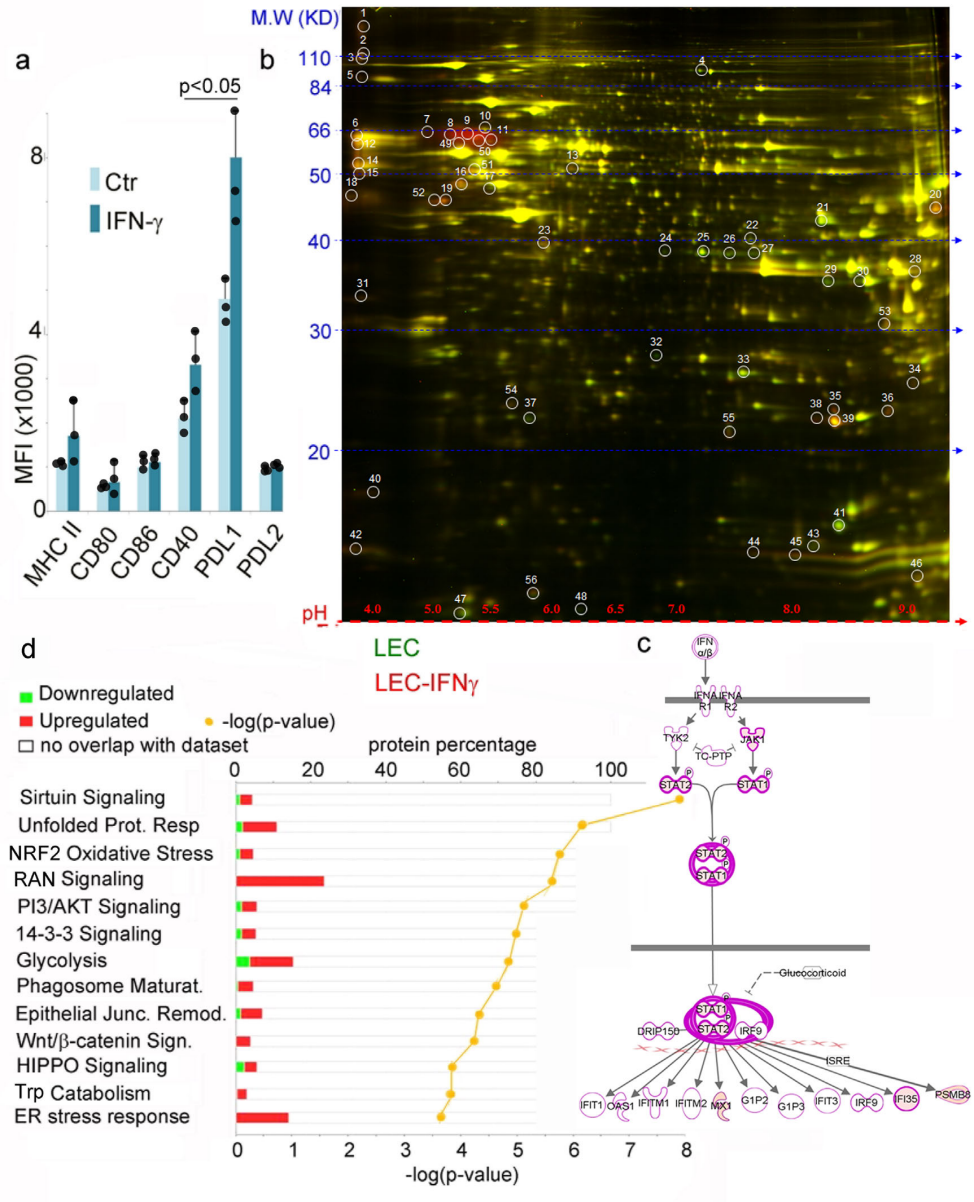
IFN-γ-stimulated LEC upregulate enzymes of tryptophan catabolism. **a** Bar graph reporting the mean fluorescence index and standard deviation of *n* = 3 biologically independent replicates of FACS analysis for MHC class II and co-stimulatory molecules present at the cell surface of LEC untreated or treated with IFN-γ (100 ng/ml for 24 h). Averages were statistically analyzed using a two-tailed paired student’s *t* test. **b** 2DIGE of cell lysates (25 μg of protein) treated as in (**a**): lysates from untreated cells were labeled with Cy3 (green) and lysates from IFNγ cells were labeled with Cy5 (red). Proteins were resolved on a 4–20% SDS-PAGE gels and gel images were acquired on a Typhoon 9400 scanner and analyzed using DeCyder Software (V6.0, GE Healthcare). Spots with a LEC/LEC IFN-γ ratio 2.5< or >2.5 were collected for MS/MS analysis (Supplementary Data 1) (*n* = 1). **c**, **d** The 2DIGE analysis (in **b**) was complemented with label-free quantitation (LFQ) proteomic analysis of *n* = 3 biologically independent replicates (triplicates of untreated or IFNγ-treated LEC as in (**b**). Data are available at ProteomeXchange Consortium via the PRIDE partner repository with the dataset identifier PXD015865. Pathway analysis of LEC proteins up- or downregulated following IFN-γ treatment. Data were analyzed with IPA (QIAGEN Inc., https://www.qiagenbioinformatics.com/products/ingenuity-pathway-analysis). For network generation, datasets containing gene identifiers (gene symbols) were uploaded into the IPA application together with their rescaled log2 transformation of protein’s area ratios, extracted from label-free quantitative (LFQ) MS1 analysis provided by the PEAKS Q module implemented in PEAKS 8.0/8.5. The probability of having a relationship between each IPA indexed biological function and the experimentally determined genes was calculated by the right-tailed built-in Fisher’s exact test. The level of significance was set to a *p*-value of <0.05. **c**, **d** IPA-predicted upregulation (in red) of inflammatory pathways in IFNγ-treated LEC, including (**c**) STAT1-mediated intracellular signaling.

**Fig. 4:**
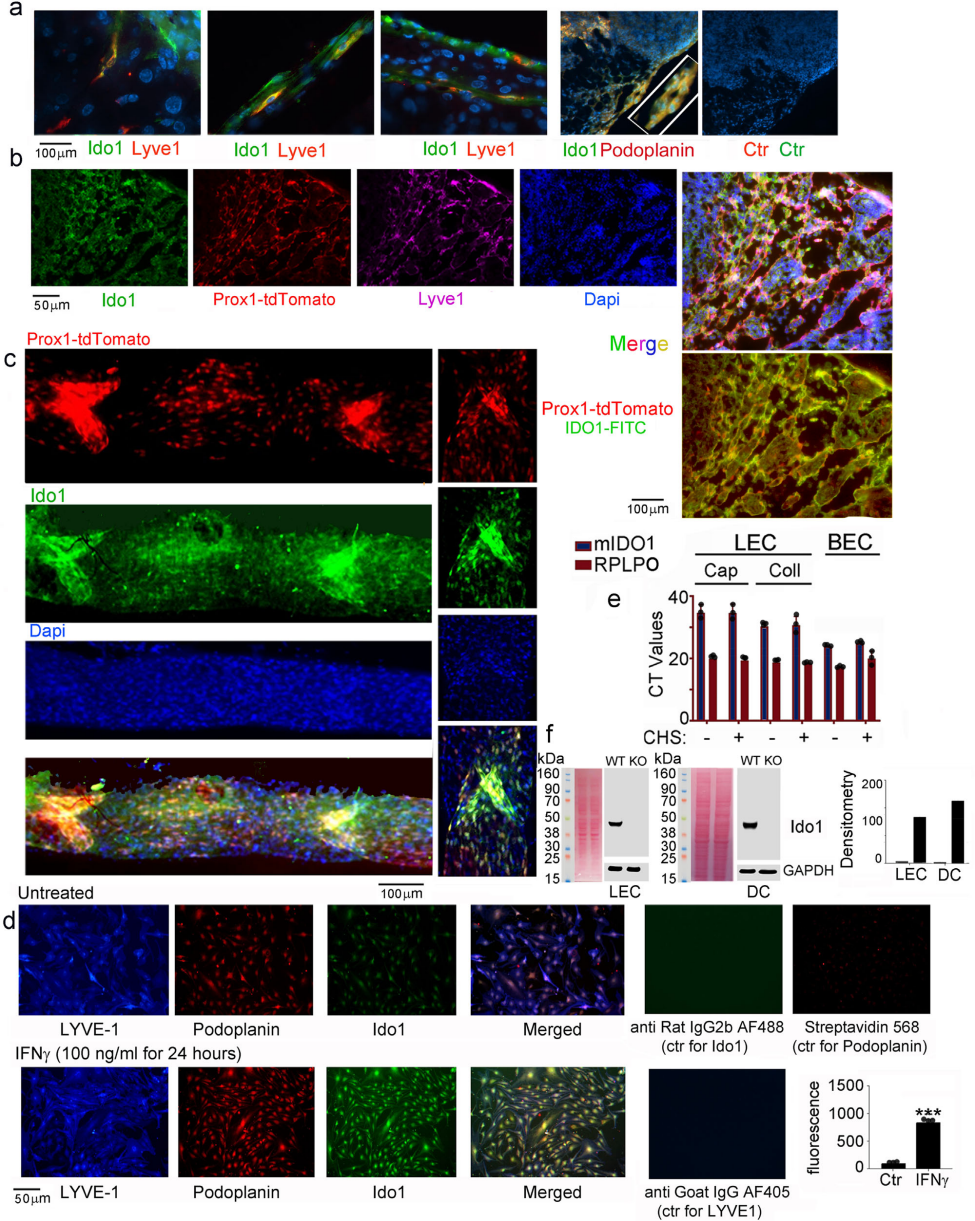
Nodal LECs express IDO1. **a**, **b** Immunofluorescence of inguinal lymph nodes harvested from Ctr or Prox1-td-Tomato mice to detect LYVE-1^+^, Podoplanin^+^ LECs, which are also positive for IDO1 staining (*n* = 3 biologically independent replicates). **c** Immunofluorescence of the thoracic duct harvested from Prox1-td-Tomato mice stained for IDO1 (*n* = 3 biologically independent replicates). Twenty-four hours prior to the harvesting mice were injected (i.p.) with 5 μg of IFN-γ. The image depicts a lymphangion, which is the lymphatic vessel area between two valves (one of the valve areas is magnified (×40)). **d** Nodal LEC were grown on 0.1% gelatin-coated glass coverslip in a petri dish and left untreated or treated ON with 100 ng/ml IFNγ). Cells were then fixed with 4% PFA and permeabilized in 0.1% Triton X-100. After blocking with 2% BSA, cells were incubated with the following conjugated primary antibodies: AF488 anti-mouse Lyve 1; PE anti-mouse podoplanin; AF488-anti-mouse CD31 (*n* = 3 biologically independent replicates). Fluorescence averages were statistically analyzed using a two-tailed paired Student’s *t* test *p* < 0.001 (***). **e** qPCR analysis of the *Ido1* mRNA transcript in LECs, collected from lymphatic capillaries (Cap) or lymphatic collectors (Coll) and blood endothelial cells (BECs). LECs and BECs were FACS-sorted from control mice or mice where contact hypersensitivity (CHS) was induced in the ear skin by topical application of 2% oxazolone (mean ± SD from biological replicates (*n* = 3), each calculated from 3 technical replicates). **f** IDO1 Western blot analysis of LEC and DC lysates harvested from wild-type and *Ido1* ko mice cultured with IFN-γ (50 ng/ml) to maximize IDO1 expression. Bar graphs represent densitometry average readings ± SD of *n* = 2 biologically independent replicates. Source data for (**a**–**f**) are provided as Source data file.

In the primary pathway (http://www.genome.jp/kegg/pathway/map/map00380.html) Trp is metabolized to *N*-formyl-kynurenine (by IDO1, IDO2, or TDO), which is then converted to kynurenine and 3-hydroxy-kynurenine, the major metabolites observed in DCs. In this pathway, 3-hydroxy-kynurenine can then be further metabolized to 3-hydroxy-anthranilic acid and quinolinic acid, or be converted to 3-HKA in the lateral pathway of Trp catabolism. Since the Trp lateral pathway, which generates 3-HKA, was never reported before in eukaryotic cells, we set to determine which cells produced 3-HKA (Fig. 2). We chose to analyze several cell types known to have one or more of the Trp metabolism limiting enzymes (IDO1, IDO2, or TDO) including dendritic cells (DCs), follicular reticular cells (FRC), and blood endothelial cells (BEC). Since 3-HKA was present in the lymphatic fluid as well, we also analyzed LEC, which are also IDO1+ as determined by 2DIGE and immunofluorescence (Figs. 3 and 4 and Supplementary Data 1). LEC were left untreated or treated for 12 or 24 h with either TGF-β (10 ng/ml), IFN-γ (50 ng/ml), or IL-4 (5 ng/ml). Supernatants were then collected and analyzed by mass spectrometry. Metabolomic analysis detected low nanomolar amount of 3-HKA in unstimulated cells which increased following IFN-γ treatment (Fig. 2d). Metabolomic analysis confirmed that 3-HKA is also produced by human nodal LEC, following IFN-γ (Fig. 2e) and that human and mouse dermal LEC produced only trace amounts of 3-HKA, even after IFN-γ or LPS stimulation (Fig. 2e, f). Additional non-professional APC, including BEC and FRC did not produce 3-HKA (Fig. 2g, h). Professional APC, such as DCs, also generated low nanomolar amount of 3-HKA (Fig. 2i). Finally, different mouse and human tumor cell lines were analyzed for production of Trp metabolites (Fig. 2j), which are known to contribute to the suppression of anti-tumor immune responses^4,5^. Among all Trp metabolites, 3-HKA was the most abundant in both mouse and human breast and lung cancer cells (Fig. 2k).

### 3-hydroxy-L-kynurenamine decreases IFN-γ-mediated DC activation

**Fig. 5:**
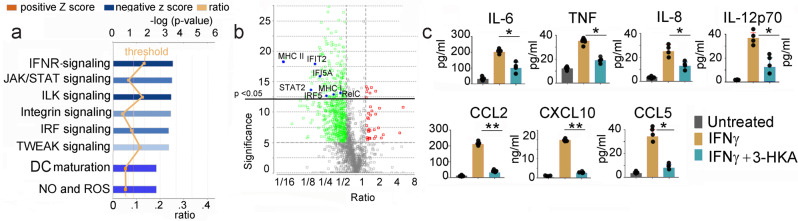
3-HKA confers immunosuppressive properties to DCs. **a** Quantitative IPA analysis depicting biochemical pathways predicted to be up- (orange) or downregulated (blue) in mouse splenic DCs following treatment with 50 ng/ml IFNγ for 24 h in presence or absence of 1 μM 3-HKA, as analyzed by label-free quantitative (LFQ) proteomics. The proteomic data are available at ProteomeXchange Consortium via the PRIDE partner repository with the dataset identifier PXD015865. For network generation, datasets containing gene identifiers (gene symbols) were uploaded into the IPA application together with their rescaled log2 transformation of average protein’s area ratios which were extracted from label-free quantitative (LFQ) MS1 analysis provided by the PEAKS Q module implemented in PEAKS 8.0/8.5. The probability of having a relationship between each IPA indexed biological function and the experimentally determined genes was calculated by the right-tailed built-in Fisher’s exact test. The level of significance was set to a *p*-value of <0.05. **b** Volcano-plot of the mouse splenic DC proteome from cells treated with 50 ng/ml IFNγ for 24 h in presence or absence of 1 μM 3-HKA (Supplementary Fig. 4a). Proteins in green are downregulated while those in red are upregulated (full proteomic data analysis is presented in Supplementary Data 2). Statistical significance (by one-way ANOVA for group comparison built-in the PEAKS X software) of *p* < 0.05 is represented by the horizontal line. **c** Quantitative analysis of pro-inflammatory chemokines and cytokines secreted by monocytes-derived human DCs treated or untreated with 1 µM 3-HKA and with 50 ng/ml IFN-γ for 24 h. Data are reported as average ± SD of *n* = 4 biologically independent replicates (each calculated from 3 technical replicates). Significance levels are reported as *p* < 0.05 (*), *p* < 0.01 (**), and *p* < 0.001 (***) (two-way ANOVA followed by Tukey’s multiple comparison test). Source data are provided as Source data file.

Since IFN-γ is the most prominent inflammatory cytokine known to activate the Trp catabolism pathway^3^ and, as shown in Fig. 2, is a powerful upregulator of 3-HKA production, we set to analyze the role of 3-HKA during IFN-γ-mediated inflammatory responses. As target cells, we chose DCs—the most powerful initiators of innate and adaptive immunity^6,7^. As such, human monocyte-derived DCs were cultured for 24 h with IFN-γ (50 ng/ml) in the presence or absence of 3-HKA at 1 μM (titration experiments indicated that this concentration is not toxic for DCs and has a long half-life (Supplementary Fig. 3a). Comparative proteomic analysis indicated downregulation of several pathways associated with DC maturation, inflammatory responses, and oxidative stress (Fig. 5a, b, Supplementary Fig. 4a and Supplementary Data 2). Analysis of the collected supernatants indicated, as expected, an increase of IFN-γ-mediated inflammatory cytokines (IL-6, IL-12p70, and TNF) (Fig. 5c) as well as an increase of several inflammatory chemokines (Fig. 5c) in the presence of IFN-γ alone. However, the concomitant culture of DCs with IFN-γ and 3-HKA significantly decreased all the inflammatory cytokines and chemokines induced by IFN-γ (Fig. 5c).

### 3-hydroxy-L-kynurenamine inhibits NF-kB activation

**Fig. 6:**
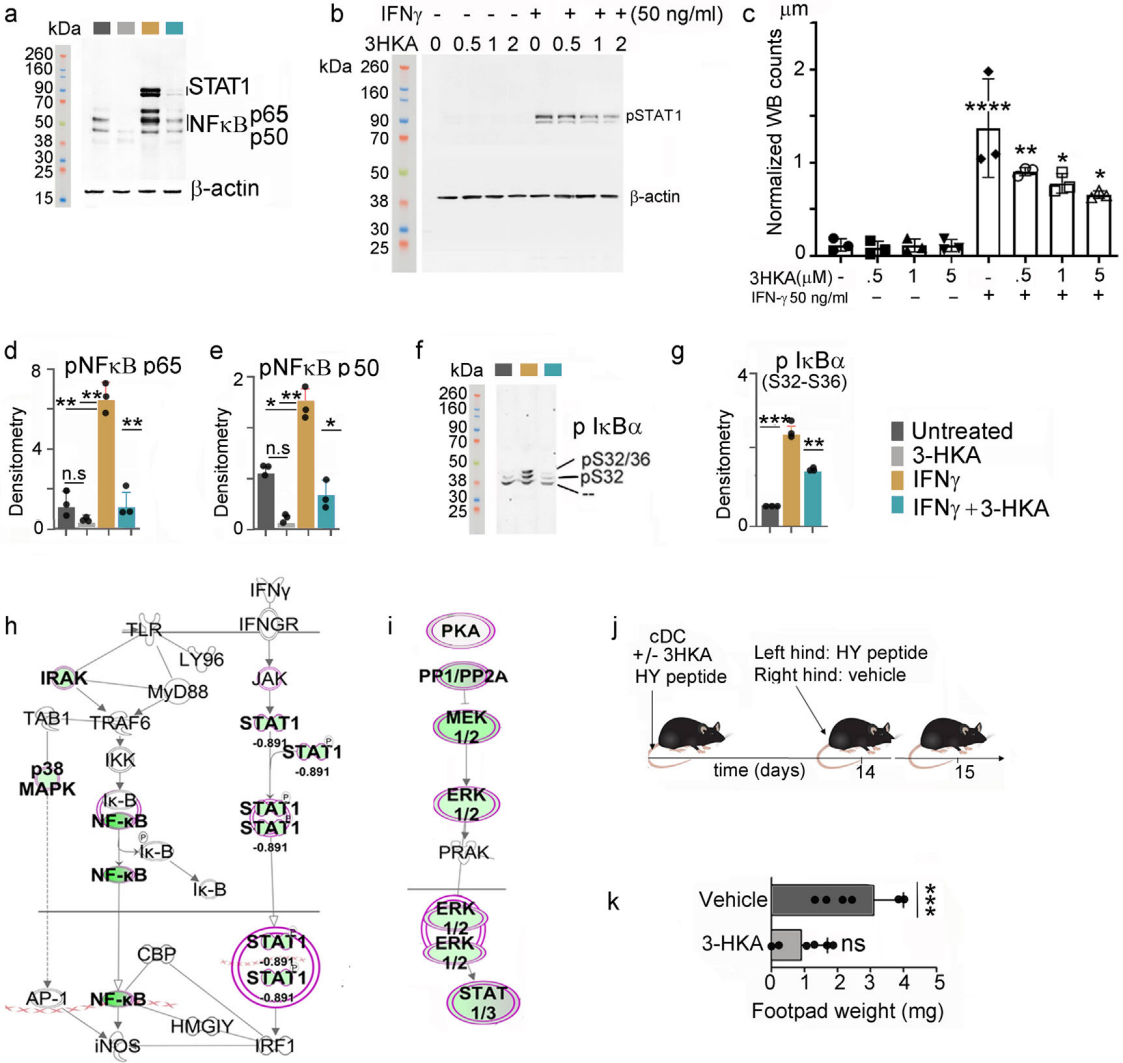
3-HKA inhibits IFN-γ-mediated STAT1/NFκB activation. **a** Representative immunoblot analysis of STAT1 and the NF-κΒ pathway-associated proteins p65 and p50 from human monocyte-derived DCs treated or untreated with 1 µM 3-HKA with or without 50 ng/ml IFN-γ for 20 min. **b** Representative immunoblot analysis of pSTAT1 from human monocyte-derived DCs treated or untreated with titrated amounts of 3-HKA with or without 50 ng/ml IFN-γ at different time points. **c**, **d**, **e** Densitometric analysis of triplicate immunoblots as reported in (**a**) and (**b**). Data of *n* = 3 biologically independent replicates are plotted as mean relative expression ± SD. Significance levels are reported as *p* < 0.05 (*), *p* < 0.01 (**), and *p* < 0.0001 (****) (two-way ANOVA followed by Tukey’s multiple comparison test). **f** Representative immunoblot analysis of pIκΒα from human monocyte-derived DCs treated or untreated with 1 µM 3-HKA with or without 50 ng/ml IFN-γ for 20 min. **g** Densitometric analysis of triplicate Western blots as reported in (**g**). Data of *n* = 3 biologically independent replicates are plotted as mean relative expression ± SD. Significance levels are reported as *p* < 0.05 (*), *p* < 0.01 (**), and *p* < 0.001 (***) (two-way ANOVA followed by Tukey’s multiple comparison test). **h**, **i** IPA-predicted quantitative decrease of proteins associated with the IFN-γ-mediated signal transduction in mouse DCs treated with 3-HKA and IFN-γ as compared with IFN-γ treatment alone (generated from data reported in Supplementary Data 2). **j** Scheme of the delayed hypersensitivity skin test assay. **k** In vivo suppression activity of HY-pulsed CD8^−^CD11c^+^ DCs (cDC) in combination with a minority fraction (5%) of the same DCs with no conditioning or conditioned in vitro with 1 µM 3-HKA for 24 h. Analysis of the delayed hypersensitivity skin reactivity of recipient mice to the eliciting peptide at 15 days is presented as change in footpad weight (experimental versus control footpads). Data are reported as average footpads weight ± SD of *n* = 6 biologically independent replicates. Data were analyzed by paired *T* test (Wilcoxon test), ***p < 0.001. Source data for (**a**–**g**) and (**k**) are provided as Source data file.

To understand where 3-HKA acts in the molecular machinery controlling secretion of pro-inflammatory cytokines, human monocyte-derived DCs, cultured under the same experimental conditions as above, were lysed to analyze the STAT1 pathway, known to be the primary pathway associated with IFN-γ stimulation^8^. After 15-min treatment, IFN-γ readily upregulated the expression of phosphorylated pSTAT1 (Y 701) (Fig. 6a–c and Supplementary Fig. 6b, c), which, upon homodimerization and nuclear translocation, binds to IFN-γ-activated sequence elements in the promoter of target genes. The upregulation of pSTAT1 was also confirmed by intracellular staining (Supplementary Fig. 4d). As expected, after 24 h of IFN-γ treatment (when pro-inflammatory cytokines are released in the media), an increase in STAT1 protein was also observed (Supplementary Fig. 4e, f). At both times, pSTAT1 and STAT1 were downregulated once 3-HKA was added to the IFN-γ-stimulated cells (Fig. 6a–c and Supplementary Fig. 4b–f). Since IFN-γ can also activate the classical NF-κΒ pathway^8^, we tested the same cell lysates for proteins of the NF-κΒ complex (p65 and p50), as well as expression and phosphorylation of its inhibitor IκΒα (Fig. 6a, d–g and Supplementary Fig. 4g–j). After 24 h treatment with IFN-γ alone, p65 and p50 were highly upregulated. However, in the presence of 3-HKA, p65 and p50 expressions decreased to almost basal levels (Fig. 6a, d, e). Moreover, phosphorylation of IκΒ, (leading to its proteasomal degradation and thus nuclear translocation of NF-κB heterodimers) was observed following 10 min of IFN-γ treatment and was decreased following 3-HKA incubation (Fig. 6f, g). Downregulation of the STAT1/NF-kB pathway, upon 3-HKA treatment, was also observed by LFQ proteomic on mouse cDC cultured for 18 h with IFN-γ in presence or absence of 3-HKA (Fig. 6h, i Supplementary Fig. 4 and Supplementary Data 2).

Altogether, our results on both mouse and human monocyte-derived DCs indicated that 3-HKA is a biogenic amine since it inhibits IFN-γ-mediated JAK-STAT1 and NF-κΒ activation, which ultimately results in reduced secretion of pro-inflammatory cytokines and chemokines (Fig. 6 and Supplementary Data 2).

### 3-hydroxy-L-kynurenamine treatment inhibits inflammation in vivo

To analyze the in vivo relevance of 3-HKA in modulating immune responses, we performed a DTH skin test assay, an established protocol for measuring the in vivo induction of antigen-specific immunoreactivity versus tolerance in DCs^9–12^. In these experiments, female C57BL/6J mice were sensitized i.p. with the HY peptide (a minor histocompatibility male antigen), presented by conventional DCs (cDCs), administered in combination with a minority fraction of the same cells (5%) conditioned with 3-HKA or medium alone for 24 h^13^ (Fig. 6j, k). Immune reactivity was assessed at 2 weeks after priming by intrafootpad challenge with the HY peptide in the absence of cDCs^13^. As expected, the default priming ability of immunostimulatory cDCs was not affected by the presence of untreated cDCs. However, DC pretreatment with 3-HKA induced a statistically significant suppression of HY-mediated reactivity (Fig. 6k) thus indicating that the biogenic amine confers an immunosuppressive phenotype on DCs detectable in vivo.

**Fig. 7:**
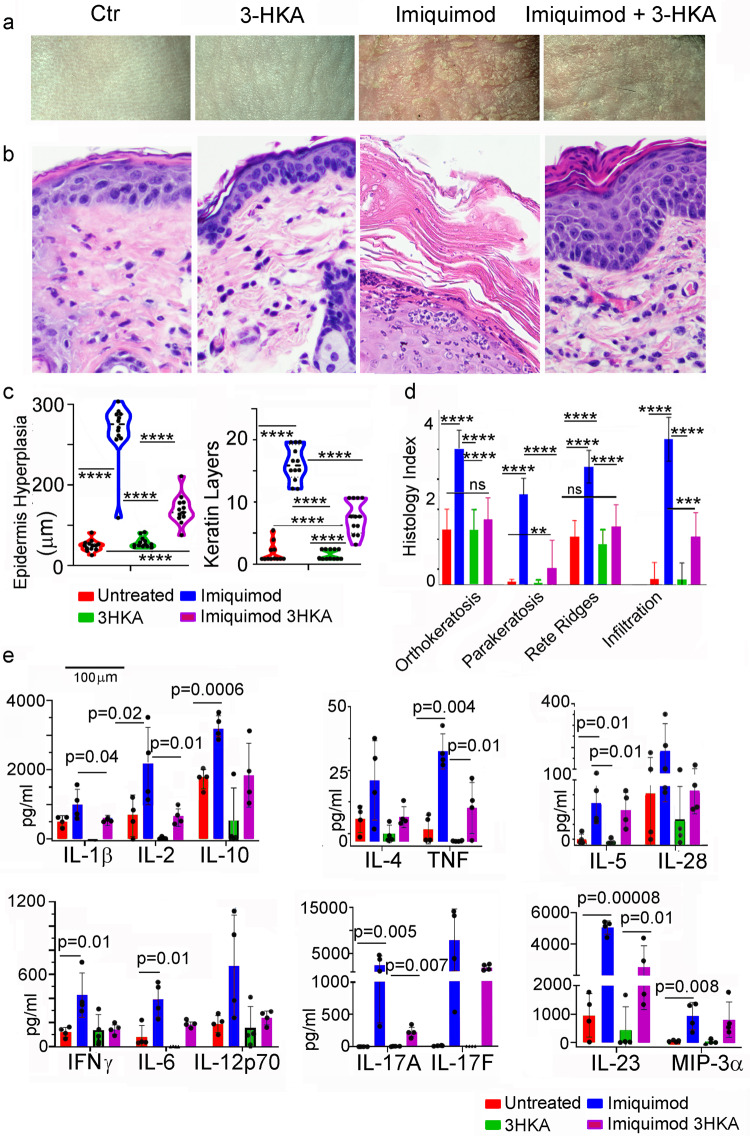
3-HKA treatment reduces psoriasis-associated skin inflammation. **a**–**d** Psoriasis was induced in C57BL/6 mice by shaving a “1 × 1” area on the mouse back and applying the TLR7 agonist imiquimod for 7 days (imiquimod). Some of the mice were also treated with 3-HKA alone (3-HKA) or in combination with imiquimod (imiquimod + 3-HKA) (3-HKA; 50 mg/kg i.p.; for 7 days). Controls are mice that were shaved but left untreated (Ctr). After 7 days, mice were sacrificed and skin harvested for histological analysis. Each skin sample was evaluated blindly by a board-certified veterinary pathologist for psoriasis-form features. Each sample was evaluated at three randomly chosen regions. *n* = 12 biologically independent replicates. **b**–**d** Features that were assessed were adapted from Baker et al.^27^ and included those of the keratin layer (orthokeratosis, parakeratosis), epidermis (hyperplasia, formation of rete ridges, ulceration), and the dermis (lymphocytic infiltration, superficial congestion, hemorrhage, necrosis). The severity of each finding was scored semi-quantitatively as follows: 0 = no lesion; 1 = minimal; 2 = mild; 3 = moderate; 4 = severe. **c**, **d** Statistical analysis refers to pooled data from *n* = 12 biologically independent replicates. Data are reported as average ± SD for each histological parameter. Significance levels are reported as *p* < 0.05 (*), *p* < 0.01 (**), and *p* < 0.001 (***) (two-way ANOVA followed by Tukey’s multiple comparison test). **e** Quantitative analysis of pro-inflammatory chemokines and cytokines present in skin lysates from the same mice reported in (**a**) using the mouse Th1/Th2/Th17 Array Q1 (Raybiotech). Data from *n* = 4 biologically independent replicates are reported as average ± SD. Significance levels are reported as *p* < 0.05 (*), *p* < 0.01 (**), and *p* < 0.001 (***) (two-way ANOVA followed by Tukey’s multiple comparison test). Source data for (**a**–**e**) are provided as Source data file.

**Fig. 8:**
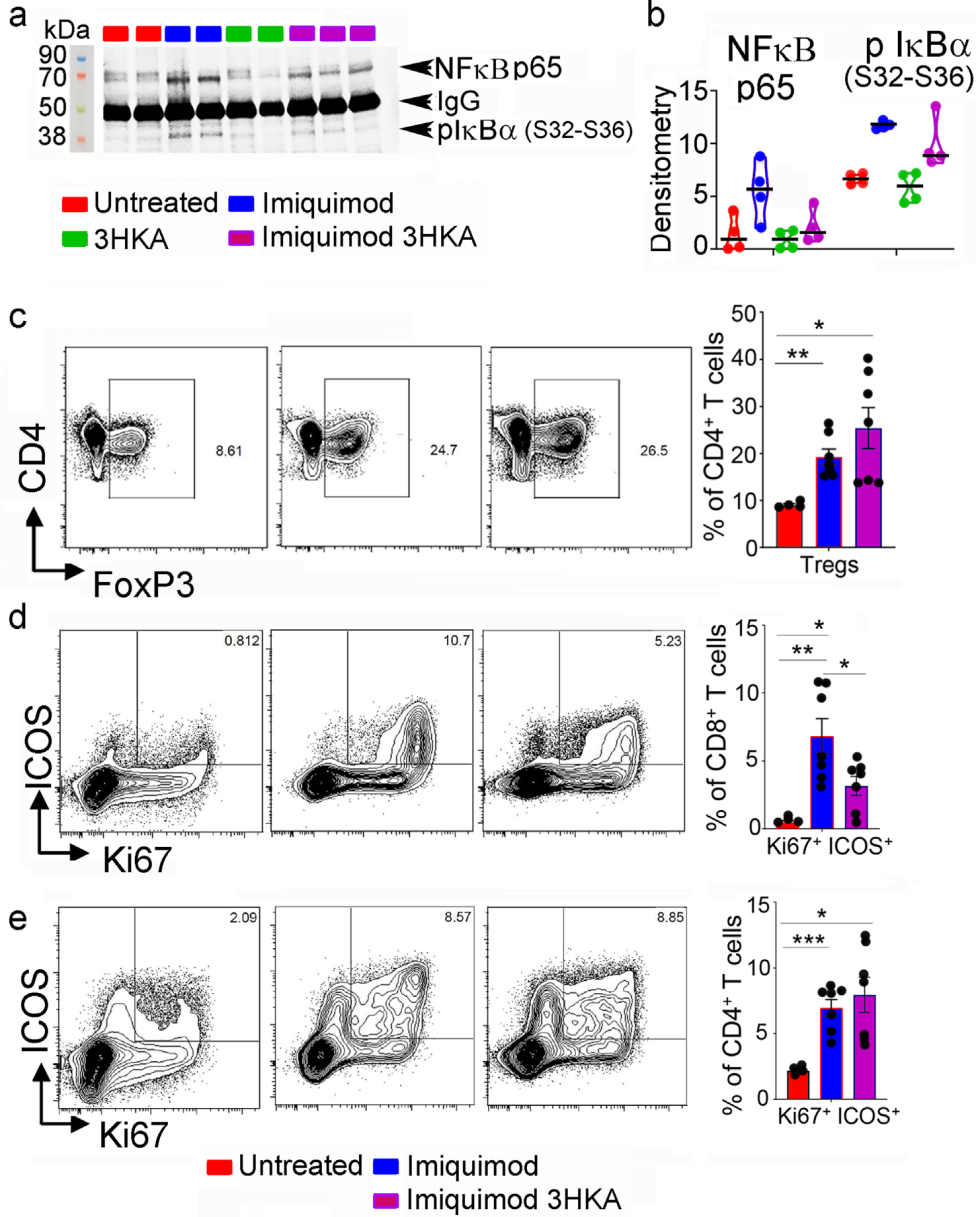
3-HKA treatment reduces inflammatory infiltration in psoriasis. **a** Representative immunoblot analysis of the NF-κΒ pathway-associated proteins p65 and pIκΒα as detected in skin samples from the same mice reported in (Fig. 7a). **b** Densitometry of immunoblot blot bands was normalized to IgG levels present in skin lysates. Data from *n* = 4 biologically independent replicates are reported as average ± SD. Significance levels are reported as *p* < 0.05 (*), *p* < 0.01 (**), and *p* < 0.001 (***) (two-way ANOVA followed by Tukey’s multiple comparison test). **c**) Flow cytometry analysis of lymph node of untreated (*n* = 4), imiquimod (*n* = 7), or 3-HKA plus imiquimod-treated C57BL/6J mice (*n* = 7). Representative plot of CD4^+^/FoxP3^+^ cells. Bar graph report % of CD4^+^/FoxP3^+^ cells for each analyzed mouse. Data are reported as average ± SEM analyzed by unpaired Student’s *t* test, two-tailed (untreated vs. imiquimod—**p = 0.0017, untreated vs. imiquimod 3-HKA—*p = 0.022, imiquimod vs imiquimod 3-HKA—ns *p* = 0.219). **d** Representative plot of ICOS^+^/Ki67^+^ CD8^+^ T cells. Bar graph report % of ICOS^+^/Ki67^+^ CD8 T cells for each analyzed mouse. Untreated (*n* = 4), imiquimod (*n* = 7), or 3-HKA plus imiquimod-treated C57BL6/J mice (*n* = 7). Data are reported as average ± SEM analyzed by unpaired student’s *t* test, two-tailed (untreated vs imiquimod—**p = 0.0054, untreated vs. imiquimod plus 3-HKA—*p = 0.021, imiquimod vs imiquimod plus 3-HKA—*p = 0.028). **e** Representative plot of ICOS^+^/Ki67^+^CD4^+^ T cells. Bar graph report % of ICOS^+^/Ki67^+^CD4^+^ T cells for each analyzed mouse. Untreated (*n* = 4), imiquimod (*n* = 7), or 3-HKA plus imiquimod-treated C57BL6/J mice (*n* = 7). Data are reported as average ± SEM analyzed by unpaired Student’s *t* test, two-tailed (untreated vs. imiquimod—***p = 0.0004, untreated vs. imiquimod plus 3-HKA—*p = 0.0112, imiquimod vs imiquimod 3-HKA—ns *p* = 0.512) (Supplementary Fig. 5). Source data for (**a**–**c**) are provided as Source data file.

The clinical relevance of 3-HKA in modulating inflammatory responses was analyzed in two well-characterized models of inflammatory diseases such as psoriasis and nephrotoxic nephritis. Psoriasis was induced following skin topical application of the TLR7 agonist imiquimod for 7 days^14^ (Fig. 7a). As expected, imiquimod-treated mice developed skin inflammation with increased thickening of the stratum corneum (hyperkeratosis, orthokeratosis, and parakeratosis), scaling, and increased rete ridges formation (Fig. 7a–d). A significant decrease in all the above parameters was observed following 3-HKA i.p. administration, throughout the same length of imiquimod treatment (Fig. 7a–d). Topical imiquimod induces mostly skin migration and activation of plasmacytoid dendritic cells (pDCs)^15,16^, which selectively express very high levels of the TLR7 and TLR9 receptors^17^ and, upon activation, produce high levels of Th1 and Th17 skewing cytokines with IL-23 as a key driver^18^. Skin samples were biopsied from each animal at three individual locations and tested for the presence of pro-inflammatory cytokines and activation of the NF-κΒ pathway. Several pro-inflammatory cytokines, including IL-1β, IL-6, IFN-γ, TNF, IL-12p70, and IL-17, were all upregulated following imiquimod application (Fig. 7e). Importantly, following in vivo 3-HKA treatment, a statistically significant reduction of all these inflammatory cytokines was observed (Fig. 7e). Similarly, the in vivo activation of the NF-κΒ pathway (as detected by p65 expression and IkBα phosphorylation) induced by imiquimod was also reduced following 3-HKA treatment (Fig. 8a, b).

The adaptive immune response to imiquimod has been very well characterized due to its therapeutic efficacy in treating different types of skin cancer. In human skin treated with topical imiquimod, recruitment of CD4^+^FoxP3^+^ Treg cells has been previously reported^19^. Similarly, in our psoriasis experiments, imiquimod significantly increased CD4^+^FoxP3^+^ Treg cell numbers. Their recruitment was not further enhanced by 3-HKA (Fig. 8c Supplementary Fig. 5). Moreover, in imiquimod-treated human skin, activation of cross-primed CD8^+^ T cells has been reported^20^, also observed in our experiments with imiquimod-treated psoriatic mice (Fig. 8d). However, when imiquimod-treated mice were administered 3-HKA, a significant reduction in the frequency, proliferation, and activation (~2-fold decrease of Ki67^+^ICOS^+^) of CD8^+^ T cells, but not CD4^+^ T cells, was observed (Fig. 8d, e and Supplementary Fig. 5). The data indicated that, following 3-HKA administration, reduced inflammation and skin pathology in psoriatic mice can also be related to the reduced (50%) frequency and proliferation of activated CD8^+^ T cells (Fig. 8c–e and Supplementary Fig. 5).

**Fig. 9:**
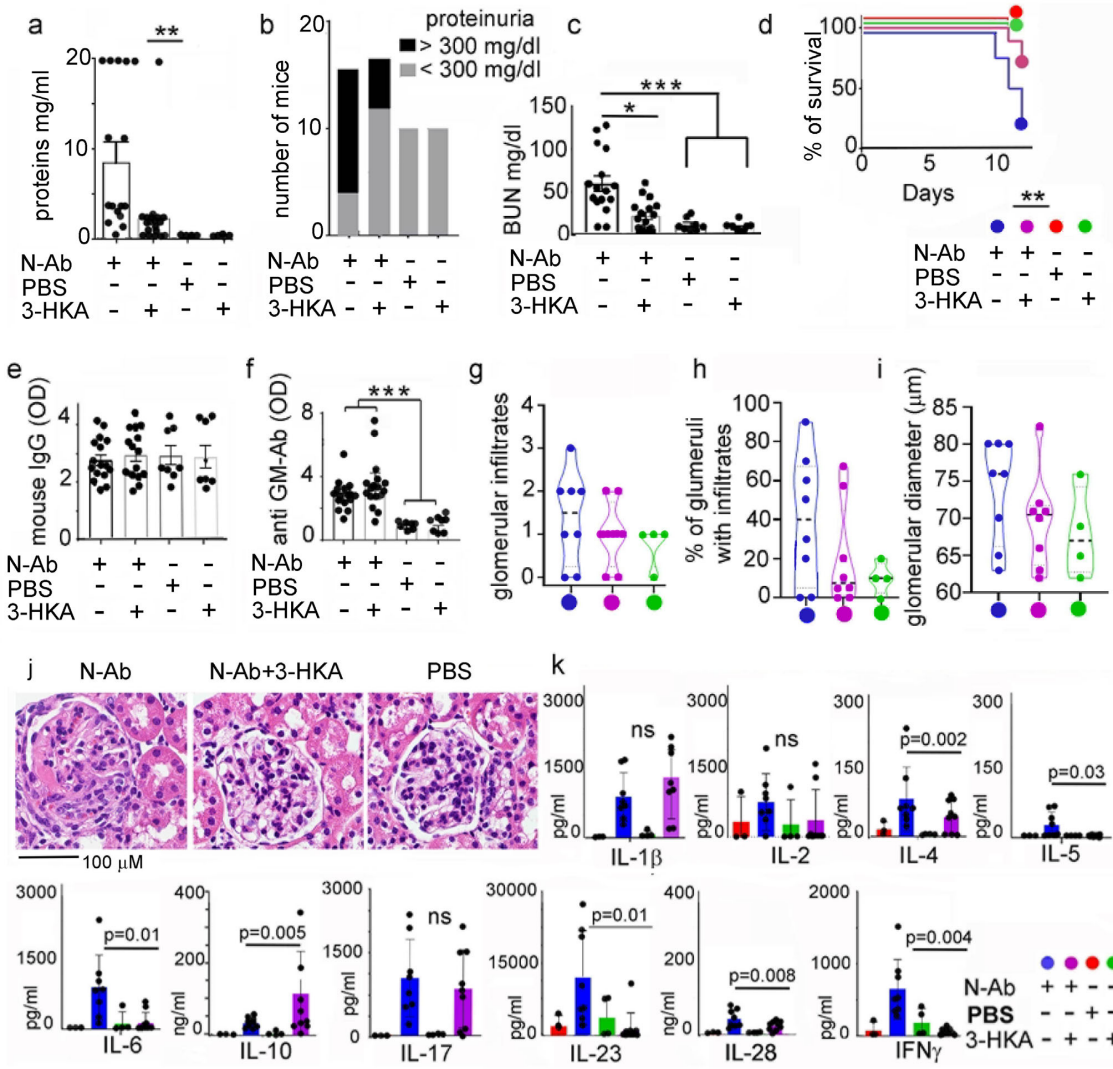
3-HKA treatment decreases nephrotoxic nephritis. **a**–**d** Nephrotoxic nephritis was induced at day 0 in 129/Sv mice as detailed in the methods. Starting at day +4, mice received daily i.p. injections of 3-HKA or vehicle (VC) control until sacrifice. N-Ab group *n* = 16; N-Ab+3-HKA group *n* = 18; PBS group *n* = 10; 3-HKA group *n* = 10. **a** Proteinuria levels at sacrifice are reported as average ± SD and statistically evaluated using two-way ANOVA followed by Tukey’s multiple comparison test. N-Ab group *n* = 16; N-Ab+3-HKA group *n* = 18; PBS group *n* = 10; 3-HKA group *n* = 10. *p* < 0.05 (*), *p* < 0.01 (**), and *p* < 0.001 (***). **b** The number of mice in each group at sacrifice that reached the predetermined cut-off value for severe proteinuria of >300 mg/dl. **c** Blood urea nitrogen (BUN) levels, measured at sacrifice, are reported as average ± SD and statistically evaluated using two-way ANOVA followed by Tukey’s multiple comparison test. N-Ab group *n* = 16; N-Ab+3-HKA group *n* = 18; PBS group *n* = 10; 3-HKA group *n* = 10. *p* < 0.05 (*), *p* < 0.01 (**), and *p* < 0.001 (***). **d** Survival curve among the different treatments. **e**, **f** All groups of mice developed similar titers of mouse anti-rabbit Ab and anti-GBM Ab. Data are reported as average ± SD and statistically evaluated using two-way ANOVA followed by Tukey’s multiple comparison test. N-Ab group *n* = 16; N-Ab+3-HKA group *n* = 18; PBS group *n* = 10; 3-HKA group *n* = 10. *p* < 0.05 (*), *p* < 0.01 (**), and *p* < 0.001 (***). **g**–**j** Each kidney was evaluated blindly by a board-certified veterinary pathologist at three randomly chosen regions. Features that were assessed included glomerular infiltrates, % of glomeruli with immune infiltrates and glomerular diameter (μm). The severity of each finding was scored semi-quantitatively as follows: 0 = no infiltration; 1 = minimal; 2 = mild; 3 = moderate; 4 = severe. N-Ab group *n* = 16; N-Ab+3-HKA group *n* = 18; PBS group *n* = 10; 3-HKA group *n* = 10. **k** Quantitative cytokine analysis in kidney lysates from the same mice reported in (**a**). Data are reported as average ± SD and evaluated using two-way ANOVA followed by Tukey’s multiple comparison test N-Ab group *n* = 8; N-Ab+3-HKA group *n* = 9; PBS group *n* = 4; 3-HKA group *n* = 4. Source data for (**g**–**k**) are provided as Source data file.

We next tested the in vivo efficacy of 3-HKA in a second animal model of inflammation; nephrotoxic nephritis, which is induced by injecting Ab against the kidney glomerular basement membrane, causing proliferative glomerulonephritis and severe kidney failure. Mice injected with nephrotoxic serum alone developed significant levels of proteinuria (Fig. 9a, b) and increased serum urea nitrogen (BUN) (Fig. 9c), consistent with the development of immune-mediated glomerulonephritis and renal dysfunction. The increase in both proteinuria and BUN following administration of the nephrotoxic challenge was attenuated with 3-HKA treatment (Fig. 9a–c). Importantly, the treatment also significantly improved the survival of mice given nephrotoxic serum (Fig. 9d). The efficacy of the 3-HKA treatment was not due to an effect on the induction phase of the disease, since the titers of the nephritogenic rabbit anti-mouse glomerular antibodies and the cross-linking mouse anti-rabbit IgG antibodies were the same in the presence or absence of 3-HKA (Fig. 9e, f). This indicated that the effects seen with 3-HKA treatment are likely due to a specific modulatory effect on kidney inflammation, rather than inhibition of nephritis development. Indeed, histological evaluation of the kidney glomeruli indicated a reduced trend of glomerular infiltrates and diameter in the 3-HKA-treated group (Fig. 9g–j). To further support this hypothesis, a significant decrease in several pro-inflammatory cytokines, most notably IL-6, and IFN-γ, was observed (Fig. 9k). These data confirmed the efficacy of 3-HKA in reducing inflammation in an additional inflammatory model.

### IDO1 knockdown in lymphatic endothelial cells increases inflammation

**Fig. 10:**
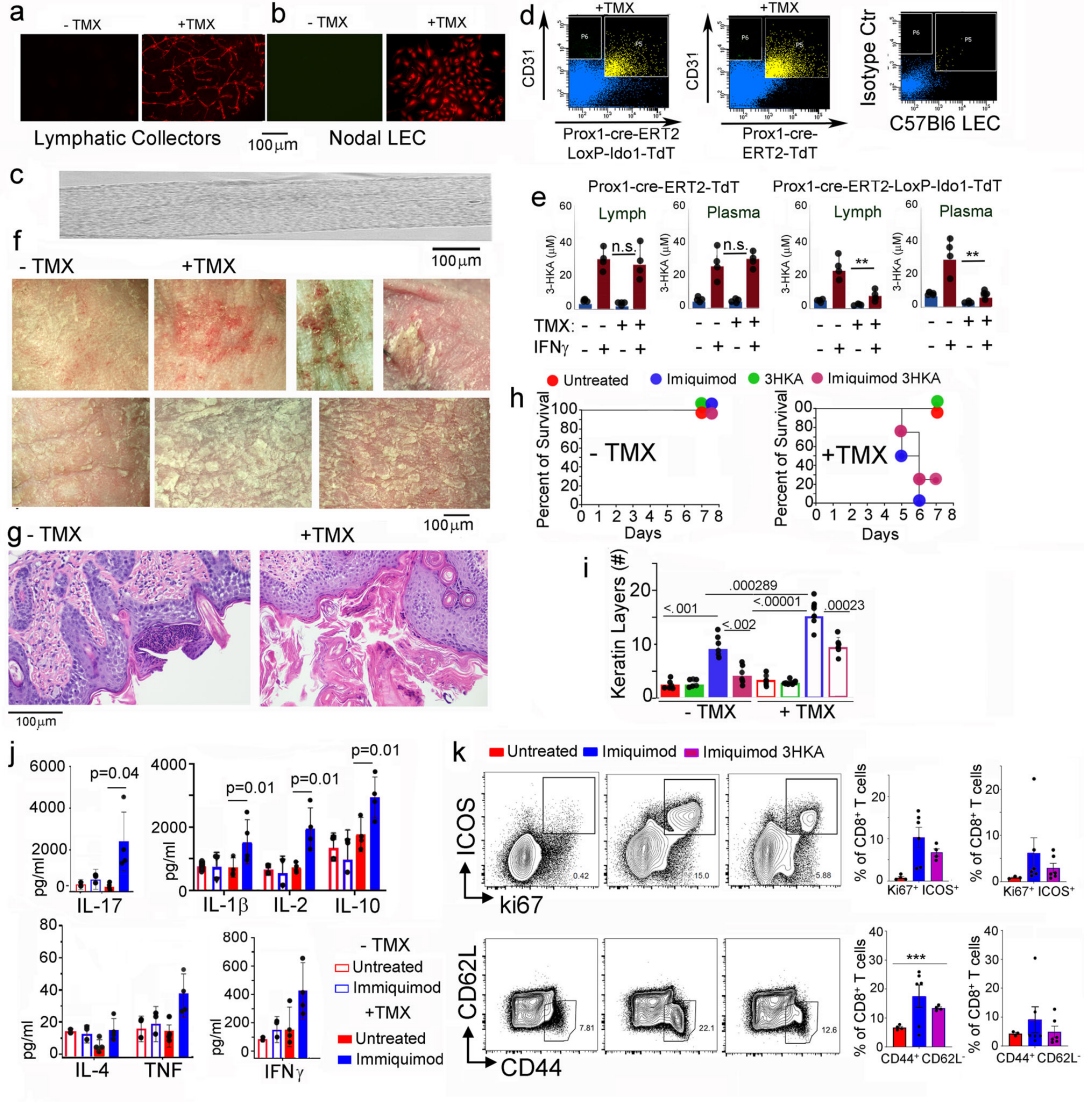
Deletion of IDO1 in lymphatic endothelial cells exacerbates psoriasis. **a** Lymphatic collectors from Prox1-cre-ERT2-TdT-LoxP-IDO1 mice, visualized after tamoxifen treatment (+TMX), vs untreated (−TMX) (*n* = 24 biologically independent replicates). **b** Nodal LECs express tdTomato only after tamoxifen treatment (+TMX) (*n* = 24 biologically independent replicates). **c** Thoracic ducts and lymph nodes were harvested from tamoxifen-treated Prox1-Cre-ERT2-TdT-LoxP-Ido1 mice and tamoxifen-treated Prox1-Cre-ERT2-TdT controls (*n* = 24 biologically independent replicates). **d** LECs were sorted from the same mice as in (**c**) using CD31 and TdTomato as markers (*n* = 12 biologically independent replicates). **e** 3-HKA quantification in the lymphatic fluid and plasma (*n* = 4 biologically independent samples) of Prox1-Cre-ERT2-TdT-LoxP-Ido1 mice and Prox1-Cre-ERT2-TdT controls treated (+TMX) or untreated (−TMX) with tamoxifen. Data are presented as average ± SD of biological quadruplicates (*n* = 4) and analyzed by two-tailed paired student’s *t* test, *p* < 0.01 (**). **f**–**i** Psoriasis was induced in the Prox1-cre-ERT2-TdT-LoxP-Ido1 as described in Fig. 7, a more severe psoriasis as indicated by (**f**, **g**, **i**) histological evaluation and (**h**) animal survival curve, of tamoxifen-treated mice (+TMX) as compared to the untreated animals (−TMX) (*n* = 12 biologically independent samples). **i** Histological quantification is presented as number of keratin layers, quantified in biological replicates ± SD (*n* = 7 biologically independent samples). Data are presented as average ± SEM analyzed by two-tailed paired student’s *t* test. **j** Quantitative analysis of pro-inflammatory chemokines and cytokines present in skin lysates from Prox1-cre-ERT2-TdT-LoxP-Ido1 mice (+TMX) and (−TMX) tamoxifen, 4 days after psoriasis induction. Data from independent biological replicates (*n* = 4) plotted as mean relative expression ± SD. Significance levels are reported as *p* < 0.05 (*), *p* < 0.01 (**), and *p* < 0.001 (***) (two-way ANOVA followed by Tukey’s multiple comparison test). **k** Flow cytometry analysis of lymph node from the same mice as in (**g**, **h**); representative plot of ICOS^+^/Ki67^+^ CD8^+^ T cells. Bar graph report % of ICOS^+^/Ki67^+^ CD8^+^ T cells for each analyzed mouse and representative plot of CD44^+^/CD62L^−^ CD8^+^ T cells. 3-HKA (*n* = 4), imiquimod (*n* = 6), or 3-HKA plus imiquimod-treated C57BL/6J mice (n = 4). Data are presented as average ± SEM analyzed by two-tailed unpaired student’s *t* test, *p* < 0.001 (***). Source data for (**a**–**c**) and (**j**–**k**) are provided as Source data file.

To evaluate the in vivo role of the IDO1 pathway in LECs anti-inflammatory activity, we crossed the Ido1 floxed mice (generous gift from M. Veldhoen) with the Prox1-Cre-ERT2-TdT (Jackson Laboratories) to achieve LEC-specific deletion of *Ido1*. Restricted expression of the Cre-recombinase in LECs was tested in each mouse by visualizing Td Tomato in the ear lymphatics before and after tamoxifen treatment (Fig. 10a, b). Prox1-Cre expression and wild type or floxed Ido1 was confirmed by qPCR, in each experimental mouse, on LECs sorted from the lymph nodes and thoracic duct of Prox1-Cre-ERT2-LoxP-Ido1-TdT mice or Prox1-Cre-ERT2-TdT controls (Fig. 10c, d). Quantification of 3-HKA in lymphatic fluid and plasma of both Cre-ERT2-LoxP-Ido1-TdT mice or Prox1-Cre-ERT2-TdT controls (plus or minus tamoxifen treatment) indicated a decrease of 3-HKA in the Cre-ERT2-LoxP-Ido1-TdT mice only, following tamoxifen treatment (Fig. 10e). In both biological fluids, 3-HKA decreased from around 20 μM to 3–5 μM, indicating that LECs are a major source of 3-HKA production (Fig. 10e).

Next, Psoriasis was induced by imiquimod treatment in tamoxifen feed (tmx+) and controls (tmx−) mice (Fig. 10f–g). Whereas no mortality was observed in the tmx− controls, 50% of tmx+ mice died by day 5 (Fig. 10h), and the rest of the animals displayed a more severe form of Psoriasis. Presence of ulcerations and plaques were observed in tmx+ mice, whereas in tmx− mice skin inflammation was still developing (Fig. 10f, g, i). Skin samples were biopsied from each animal at three individual locations and tested for the presence of pro-inflammatory cytokines. Whereas in tmx− mice no significant increase of pro-inflammatory cytokines could be detected at this early stage of the disease (day +4 after induction), in tmx+ mice a significant increase of IL-1β, IFN-γ, TNF, and IL-17 could already be measured (Fig. 10j). In addition, in tmx+ mice, the inhibitory effect of 3-HKA on the development of effector CD8^+^ T cells was reduced (Fig. 10k and Supplementary Fig. 6), likely due to the strong inflammatory response elicited by inhibition of the IDO1 pathway.

Biogenic amines are biologically active substances containing one or more amine groups that are mainly formed from decarboxylation of amino acids. Up until now, only serotonin and melatonin have been characterized as biogenic amines produced by Trp catabolism pathways not involving IDO1. Herein, we report a third, previously uncharacterized biogenic amine, 3-HKA, which is released by both professional and non-professional antigen-presenting cells during inflammatory conditions and spontaneously released by all the cancer cells tested so far. This metabolite has in vitro and in vivo anti-inflammatory effects by inhibiting the IFN-γ-mediated STAT1/NF-κΒ pathways, which translate into decreased biochemical, cellular, histological, and clinical signs of experimental psoriasis and nephrotoxic nephritis. In both models, 3-HKA also decreased CD8^+^ T-cell proliferation and naive-to-effector CD8^+^ cell maturation, overall controlling immune activation during inflammatory conditions. Therefore, we propose that this biological amine is an important component of LEC-mediated immune tolerance.

In addition, the fact that in all examined cancer cell lines 3-HKA was the major Trp metabolite also indicated that antagonist to this amine may have therapeutic applications in cancer immunotherapy.

## Methods

### Reagents

Trifluoroacetic acid (cat# T6508), iodacetamide (cat# I1149-5G), RIPA buffer (cat# R0278), Triton X-100 (cat# 9002-93-1), TCEP-HCL (Tris (2-carboxyethyl) phosphine hydrochloride) (cat# 580567), iodoacetamide (cat# I1149-5G), acetone (cat# 1179124), dansylchloride (5-(Dimethylamino) naphthalene-1-sulfonyl chloride, DNSCl) (cat# D2625-1G), phosphatase inhibitor cocktail 2 (cat# P5726), phosphatase inhibitor cocktail 3 (cat# P0044), MILLIPLEX MAP lysis buffer (cat# 43-040), CHAPS, molecular biology grade (cat# 220201), sodium chloride (NaCl) (cat# S7653-250G), sodium citrate (cat# W302600-1KG-K), fetal bovine serum (FCS) (cat# F0926), magnesium chloride (MgCl_2_) (cat#M9272), glycerol (cat# G5516), and beta-glycerophosphate, disodium salt, pentahydrate (cat# 35675) were all purchased from Millipore-SIGMA-Aldrich (St. Louis, MO, USA). Acetonitrile Optima™ LC/MS (cat# A955-4), acetic acid (cat# A38S-500), formic acid Optima™ LC/MS (cat# A11710X1-AMP), HEPES Buffer (cat# BP299-100), methanol Optima™ LC/MS (cat# A456-500), thiourea (cat# AAA1282836), and Tris-HCl (cat# BP153-500) were all purchased from Fisher Scientific (Pittsburgh, PA, USA). Urea (cat# 29700), dithiothreitol (DTT) (cat# 20290), EDTA ultrapure 0.5 M solution, pH 8.0 (cat# 15575020) beta-mercaptoethanol (thioglycol) (cat# 35602BID), ammonium bicarbonate (cat# BP2413500), potassium chloride (cat# AAA116620I), potassium phosphate monobasic (cat# BP362-500), phosphoric acid (cat# 02-003-602), gel silver staining kit for mass spectrometry (cat# 24600), Novex 4–20%, Tris-Glycine Mini Gels (cat# XV04200PK20), HPLC-grade water (cat# TS-51140), and micro BCA protein assay kit (cat# 23235) were all purchased from ThermoFisher Scientific (Waltham, MA USA). Complete Proteinase inhibitor cocktail (cat# 04693116001) was purchased from Roche. Trypsin (cat# V5111), Lys-C (cat# VA1170), and GluC (cat# V1651), all sequencing grade purity were purchased from Promega (Madison, WI, USA).

### Synthesis of 3-HKA dihydroiodide and dihydrobromide

The synthesis of 3-amino-1-(2-amino-3-hydroxyphenyl)propan-1-one dihydroiodide was prepared using a slightly modified version of the procedure previously reported^21^. The starting material, i.e., 2-nitro-3-methoxybenzaldehyde commercially available by Acros, was converted in 2-(3-(2-amino-3-methoxyphenyl)-3-oxopropyl) isoindoline-1,3-dione. The subsequent reaction with HI in presence of phosphorous, under reflux in acetic anhydride for 12 h, was carried out to remove the methyl group^22^. The compound was tested for purity and structural integrity using HPLC and ^1^H-NMR. H-3KA purity was above 96%. ^1^H-NMR (400 MHz*, d*_*6*_-DMSO) δ: 3.2 (m, 2H), 3.29 (t, *J* = 6.4 Hz, 2H), 4.59 (BS, 2H), 6.49 (pt, *J* = 8.05 Hz, *J* = 7.73 Hz, 1H), 6.85 (d, *J* = 7.9 Hz, 1H), 7.22 (d, *J* = 8.3 Hz, 1H), 765 (bs, 1H), 9.85 (bs, 1H).

### Mice

Eight- to twelve-week-old C57BL/6, and B6.129-*Ido1*^*tm1Alm*^/ mice (50% male and 50% females) were obtained from Jackson Laboratory (Stock #: 000664 and Stock #: 005867). Mice were housed in an environmentally controlled condition with a 12 h light/dark cycle and allowed free access to food and water. Experimental protocols used in this study were approved by the Weill-Cornell Medicine Committee for Animal Experiments (WCM IACUC Protocol #: 2019-0024). All experiments are also compliant with all relevant ethical regulations for animal testing and research.

#### Primary cells

C57BL/6J primary lymphatic endothelial cells (LEC) and mouse primary spleen endothelial cells (BEC) (Cell Biologics, cat# C57-6092 and cat# C57-6057) were grown in tissue culture flasks pre-coated with 0.1% gelatin in mouse complete endothelial growth cell medium (Cell Biologics, cat# M1168). In all experiments, LECs and BECs were used between passages 3 and 5. LECs and BECs were seeded in complete endothelial cell medium (Cell Biologics, cat# M1168) and left untreated or treated with recombinant murine INF-γ (Peprotech; cat# 315-05).

To obtain primary fibroblast reticular cells (FRC) lymph nodes (axillary, inguinal, mesenteric, and brachial) were collected from C57BL/6J mice and digested with 0.8 mg/ml Dispase (Worthington Biochemical corporation, cat# LS02100, 0.2 mg/ml Collagenase P (Roche, cat# 11213857 001) and 0.1 mg/ml DNase I (Millipore Sigma, cat# 260913-10MU) at 37 °C on a shaker for 60 min. At 15-min intervals, digested tissue was pipetted to facility dissociation and dispersed single cells were collected. For total stromal cell culture and expansion, following lymph node digestion, cell suspension was placed in tissue culture dish with 10% FBS-DMEM for 24 h, nonadherent cells were then removed and fresh medium added, culturing was continued until confluence. After 5–10-day culture, adherent cells, consisting of 5–10% CD11b^+^ myeloid cells and ~90% CD11b^−^CD45^−^ stromal cells, were harvested and purified by FACsorting CD45^−^CD11b^−^CD31^−^gp38^+^ FRCs with a purity of >95% (CD31 AF488 Biolegend, cat# 102513 dilution 1:100; gp38 PE Biolegend, cat# 127407, dilution 1:200; CD45 APC eBioscience, cat#17-0451-82, dilution 1:200; CD11b PE Cy7 eBioscience, cat# 25-0112-82, dilution 1:100). Sorted purified FRCs were cultured in complete DMEM medium and treated overnight with or without murine IFN-γ 100 μg/ml.

### Primary human monocyte-derived dendritic cells

Peripheral blood mononuclear cells (PBMC) were purified from heparinized peripheral blood collected from healthy donors (New York Blood Bank) using Ficoll^®^-Paque PREMIUM 1.084 (Sigma-Aldrich, cat# GE17-5446-02) density gradient centrifugation. Monocyte subpopulations were further purified by separation on magnetic beads coated with human CD14^+^ antibodies (CD14 MicroBeads, human, Miltenyi Biotec, cat# 130-050-201) using the manufacturer’s instructions. Monocytes were cultured for 6 days in complete RPMI 1640 medium supplemented with 10% fetal calf serum (FCS) and 2 mM L-glutamine (Millipore-SIGMA-Aldrich, cat# SLM-240), supplemented with 50 U/mL penicillin and 50 μg/mL streptomycin (Gibco-ThermoFisher, cat# 10378-016), 10 mM HEPES (N-2-hydroxyethylpiperazine-N’-2-ethanesulfonic acid) (Fisher Scientific, cat# BP299-100) and 0.1 mM nonessential amino acids (Gibco-ThermoFisher, 11140-050). Cells were cultured at 10 × 10^6^ cells/5 mL/well in 6-well tissue culture plates (VWR, International LLC, Radnor, PA, USA, cat# 10861-696) with 20 ng/mL recombinant human IL-4 (cat# AF-200-04) and 20 ng/mL recombinant human GM-CSF (cat# 300-03), both from PEPROTECH (Cranbury, NJ, USA).

### Preparation of cell lysates

For all proteomic experiments and WB analysis, cells were lysed on ice with cold lysis buffer consisting of 150 mM NaCl, 10 mM MgCl_2_, 2 mM EDTA, 10% glycerol, 1% Triton X-100, 2.5 mM glycerophosphate, 1 mM DTT, and supplemented with complete protease and phosphatase inhibitors described in the “Reagents” section. The lysates were shaken on a rocker at 4 °C for 15 min followed by treatment with benzonase nuclease (5 units/μl lysate) on ice, for 30 min before being spun at 13,000 × *g* in a microcentrifuge for 15 min, at 4 °C. The supernatant was further cleared with EMD Millipore filters (cat# UFC 0DV 25). Quantitation of total protein from different cell lysates was performed using the Micro BCA™ Protein Assay Kit (cat# 23235 from Thermofisher Scientific). The lysates were spun at 13,000 × *g* in a microcentrifuge for 10 min, at 4 °C and the supernatants were collected. Protein quantification was performed using Pierce^TM^ BCA Protein Assay kit (ThermoFisher Scientific cat# 23225). Independent cell lysates were produced using the MILLIPLEX MAP lysis buffer (cat# S30043-MXA), for multiplex cell signaling assays used to monitor the phosphorylations on STAT and NF-κb related proteins.

### Western blot analysis

Protein lysates were mixed with the sample buffer, heated at 95 °C for 5 min, and run on a 4–20% gradient acrylamide SDS (PAGE) gels (Novex, Tris-Glycine Mini Gels, cat# XV04205PK20, from ThermoFisher Scientific) at 160 V constant following the manufacturer protocols. Proteins were transferred to the nitrocellulose membrane (0.45 μm, ThermoFisher Scientific cat# 88014) using standard wet blot procedures. Membranes were blocked in 5% nonfat dry milk (Biorad blotting-grade blocker cat# 170-6404) in 1XPBST (0.05% Tween in 1XPBS buffer, Millipore Sigma cat# P9416 and Millipore Sigma cat# 11666789001, respectively), for 1 h at room temperature. Membranes were then incubated overnight at 4 °C with the following primary antibodies: IDO1 clone 2E2 (dilution 1:2000) (Enzo Lifescience, cat# ALX-804-902-0100); IDO2 (dilution 1:500) (Santa Cruz, cat# sc-374159); anti-human STAT1 (dilution 1:1000), which detects both STAT1 alpha (91 kDa) and STAT1 beta (84 kDa) isoforms (CST; cat# 9172); anti-human phospho-Stat1 (Tyr701) (dilution 1:1000) (CST; cat# 7649) (MW 91 and 84 kDa for phospho STAT1); anti-human NF-κB p65 (dilution 1:800), (CST; cat# 8242), (MW 65 kDa for NF-kB p65); anti-human phospho-NF-κB p65 (Ser536) (dilution 1:800), (CST; cat# 3031), (MW 65 kDa for Phospho-NF-κB p65); anti-human phospho-IκBα (Ser32) (dilution 1:800) (CST; cat# 2859), (MW 40 kDa for phospho-IκBα); anti-human phospho-IκBα (Ser32/36) (dilution 1:1000) (CST; cat# 9246), (MW 40 kDa for phospho-IκBα); anti-human IκBα (dilution 1:1000) (CST; cat# 4812), (MW 39 kDa for IκBα); anti-human phospho-p44/42 MAPK (Erk1/2) (Thr202/Tyr204) (197G2) (dilution 1:800) (CST; cat# 4377), (MW 42, 44 kDa for p-Erk1/2). Equal sample loading was determined using the goat polyclonal anti-beta-actin antibody (dilution 1:1000) (Abcam, cat# ab8229) and GAPDH (Sigma, cat# G9545, dilution 1:2000).

Blots were then washed in 1X PBST and incubated at room temperature for 2 h with the following secondary Ab: Goat anti-rat IgG (ThermoFisher; cat# 31470, dilution 1:2000); goat anti-rabbit IgG HRP conjugate (Southern Biotech; cat# 4055-05, dilution 1:2000); goat anti-rabbit IgG HRP-conjugated (R&D Systems; cat# HAF008, dilution 1:1000); goat anti-mouse (Southern Biotech; cat# 1031-05, dilution 1:2000), bovine anti-goat IgG HRP (Santa Cruz Biotechnology, cat# sc2350, dilution 1:1000). The enhanced chemiluminescence assay containing Supersignal West-Pico PLUS chemiluminescence substrate (Thermo Scientific Pierce; cat# 34577) was used to develop the membranes. Densitometry analysis was performed with the ImageJ 1.80 112 software. The relative WB counts corresponding to protein expression were calculated after normalizing the individual mean gray values with respect to the gray values for beta-actin.

### 2D-DIGE expression profiling of whole protein extracts from LECs

Twenty-five micrograms of total proteins from IFN-γ-treated (50 ng/ml) or untreated LECs were labeled with 400 pmol of either Cy3 (green) or Cy5 (red) (Amersham-Fisher Scientific CyDye™ DIGE Fluor Minimal Labeling Kit, cat# 45-002-256) for 30 min on ice. The labeling reaction was stopped with 10 mM lysine (provided by the kit described above). Cy3- and Cy5-labeled proteins were mixed in 7 M urea, 2 M thiourea, 4% CHAPS, 40 mM dithiothreitol (described in the “Reagents” section), 1% IPG buffer (pH 4–7) (ThermoFisher, ZOOM® Carrier Ampholytes 4-7, cat# ZM002), 0.002% bromophenol blue (MILLIPORE-Sigma-Aldrich, cat# B0126-25G) and applied to Immobiline DryStrips (24 cm, pH 4–7; ThermoFisher, cat# ZM0012). Isoelectric focusing was performed with an Ettan IPGphor II apparatus (Cytiva, cat# 11003364). After isoelectric focusing the proteins were reduced and alkylated by successive 15-min treatments with equilibration buffer containing 2% dithiothreitol followed by 2.5% iodoacetamide (described in the “Reagents” section). Proteins were then resolved in 4–20% SDS-PAGE gels, using an Ettan DALTsix instrument (Cytiva, cat# 80648527). Gel images were acquired on a Typhoon 9400 scanner (Amersham Biosciences/GE Healthcare, Product ID: Typhoon 9400) and analyzed using DeCyder Software (V6.0, GE Healthcare). Proteins with an untreated/treated ratio of ±2.5 were excised for MS/MS analysis as described in the two sections below.

#### Sample preparation for proteomic analysis

Equal amounts of whole-cell lysates (between 30 and 100 μg) from LECs and mouse or human primary DCs, previously subjected to different treatments, were further fractionated by 2D-DIGE or by 1D SDS-PAGE as detailed above. Gels were silver-stained (Thermo Scientific, kit for mass spectrometry (cat# 24600)) and protein gel bands were cut across each sample lane. *In gel*, proteins were reduced with 25 mM TCEP.HCl in 50 mM ammonium bicarbonate buffer, containing 8 M urea at pH 8.5 for 35 min at RT followed by alkylation with 100 mM iodacetamide for 50 min in the dark at RT (chemicals described in the “Reagents” section).

*In gel* protein digestion was carried out at 37 °C overnight (12 h) in 50 mM ammonium bicarbonate buffer (pH 8.9) using a combination of 3 enzymes (25 ng/μl of trypsin/Lys-C and 10 ng/μl of GluC) for each gel piece followed by quenching with formic acid (0.2% final concentration) (chemicals and enzymes described in the “Reagents” section). The peptide mixture, extracted from all enzymatic digestions, was desalted on C18 Prep clean columns (ThermoFisher Scientific, Pierce C18 Peptide Desalting Spin Columns, cat# 89852).

### nanoLC MS/MS

Technical triplicates of the peptide mixtures were analyzed on a Q Exactive HF quadrupole orbitrap mass spectrometer (ThermoFisher Scientific, Waltham, MA, USA) coupled to an Easy nLC 1000 UHPLC (ThermoFisher Scientific) through a nanoelectrospray ion source. Peptides were separated by a 120 min linear gradient containing 4–30% acetonitrile 0.1% formic acid at 400 nL/min (Optima™ LC/MS, Fisher Scientific, Pittsburgh). Ionization was run in the positive ion mode, applying the data-dependent acquisition (DDA) mode, where full MS scans were obtained with a range of *m*/*z* from 300 to 1600, a mass resolution of 120,000 at *m*/*z* 200, and a target value of 1.00E+06 with the maximum injection time of 50 ms. HCD collision was performed on the 15 most significant peaks, and tandem mass spectra were acquired at a mass resolution of 30,000 at *m*/*z* 200 and a target value of 1.00E+05. The normalized collision energy was 32%. We excluded precursor ions with single, unassigned, or five and higher charge states from fragmentation selection.

### Protein identification

In the case of mouse LECs and primary DCs, the raw files from each technical and biological replicate were filtered, de novo sequenced, and assigned with a protein ID using Peaks 8.0/8.5 software (Bioinformatics Solutions, Waterloo, Canada), by searching against the mouse *(Mus musculus)* Swiss-Prot database (March 2018; 91,343 entries). The following search parameters were applied for the protein ID analysis: trypsin, Lys-C, and GluC were chosen for restriction enzymes with two allowed missed cleaved at one or both peptide end. The parent mass tolerance was set to 15 ppm using monoisotopic mass, and fragment ion mass tolerance was set to 0.05 Da. Carbamidomethyl cysteine (+57.0215 on C) was specified in PEAKS 8.5 as a fixed modification. Methionine, lysine, proline, arginine, cysteine, and asparagine oxidations (+15.99 on CKMNPR), deamidation of asparagine and glutamine (NQ-0.98) and pyro-Glu from glutamine (Q-18.01 N-term) were set as variable modifications. Data were validated using the false discovery rate (FDR) method built-in PEAKS 8.0/8.5 and protein identifications were accepted if they could be characterized with a confidence score of (−10lgP) 15 and above for peptides and (−10lgP) 15 and above for proteins. Searches for protein ID were retrieved using a minimum of 1 peptide per protein, after data were filtered for <1.5% FDR for peptides and <1.5% FDR for protein identifications (*p* < 0.05). An independent validation of the MS/MS-based peptides and protein identification was performed with the Scaffold (version Scaffold_4.6.2, Proteome Software Inc.) using the compatible “.mzid” files exported from PEAKS 8.0/8.5. The Scaffold built-in option “MuDPIT” was used to combine multiple files from biological and/or technical replicates. Peptide identifications were accepted if they could be established at >95.0% probability by the Peptide Prophet algorithm with Scaffold delta-mass correction. Protein identifications were accepted if they could be established at >95.0% probability. Proteins that contained similar peptides and could not be differentiated based on MS/MS analysis alone were grouped to satisfy the principles of parsimony.

#### Gene ontology annotations and analysis of networks and cellular pathways

Comparison of networks, functional analyses, biochemical and cellular pathways associated with changes in the protein expression profiles in the LECs and mouse primary DCs in response to each treatment were generated by employing the ingenuity-pathway analysis (IPA; Ingenuity Systems, Redwood City, CA, USA). For network generation, datasets containing gene identifiers (gene symbols) were uploaded into the IPA application together with their rescaled log2 transformation of protein’s area ratios, extracted from label-free quantitative (LFQ) MS1 analysis provided by the PEAKS Q module implemented in PEAKS 8.0/8.5. In the case of 2D-DIGE, the protein expression fold exported from DeCyder Software was used to perform the quantitative pathway analysis. The networks of qualified molecules were then algorithmically generated based on their connectivity index using the built-in IPA algorithm. The probability of having a relationship between each IPA indexed biological function and the experimentally determined genes were calculated by the right-tailed built-in Fisher’s exact test. The level of significance was set to a *P*-value of <0.05. Accordingly, the IPA analysis identified the molecular and cellular pathways from the IPA knowledge library of established pathways that were most significant to the dataset (−log(*p*-value) > 2.0). For the quantitative analysis of the expression profiles, IPA assigned the “*z*-score” function to all eligible canonical and cellular pathways (where a “*z* < −2” represents significant downregulation while a *z* > 2.0 represents a significant upregulation of the selected pathway).

The mass spectrometry proteomics data have been deposited to the ProteomeXchange Consortium via the PRIDE partner repository with the dataset identifier PXD015865.

The enriched multi-pathways proteomic samples, processed with PTMScan® Multi-Pathway Enrichment Kit (Cell Signaling Technology’s (CST) (#75676) were additionally analyzed with the “ssGSEA” method from REACTOME 7.6 server (https://www.reactome.org) to extract the quantitative protein expression profiles associated with different intracellular signaling pathways. Similarly, with IPA analysis, the log2 transformation of protein’s area ratios. provided by LFQ analysis of MS1 areas in PEAKS software was imported into the ssGSEA to perform the gene set variation analysis.

### Metabolomic analysis

Metabolomic analyses of tryptophan metabolites were performed as follows^23^: samples were extracted from pelleted 12–20 million LEC in an ice-cold methanol:acetonitrile:water (5:3:2 v/v) solution (described in the “Reagents” section) and then vortexed for 30 min at 4 °C. Insoluble proteins were pelleted by centrifugation (10 min at 4 °C and 12,000 × *g*) and supernatants were collected and stored at −80 °C until analysis. Human and mouse cancer cell lines were trypsined and washed twice with 1X PBS (Millipore Sigma, cat# 11666789001). Then, cells were spun for 10 min 4 °C at 14,000 rpm. The dry pellets were snap-frozen in liquid nitrogen. Extracts were thus further spun in a Speedvac until dry and resuspended in 0.1% formic acid in water. This approach allows to improve chromatographic separation and signal intensity of metabolites in the kynurenine pathway, as previously validated in technical notes^24^. Ultra-high pressure liquid chromatography coupled to mass spectrometry (UHPLC-MS) analyses for relative quantitation of Trp metabolites were performed using a Vanquish UHPLC system coupled online to a high-resolution Q Exactive mass spectrometer (ThermoFisher, Bremen, Germany). Samples were resolved over a Kinetex C18 column (2.1 × 150 mm, 1.7 µm; Phenomenex, Torrance, CA, USA, cat# 00F-4475-AN) at 45 °C. A volume of 10 μl of sample extracts was injected into the UHPLC-MS. A volume of 10 μl of each sample extract was injected and run using a 5 min gradient at 450 µL/min from 5 to 95% B (A: water/0.1% formic acid; B: acetonitrile/0.1% formic acid) and the MS was operated in positive mode. The UHPLC system was coupled online with a Q Exactive (Thermo, San Jose, CA, USA) scanning in Full MS mode at 70,000 resolution in the 60–900 *m*/*z* range, 4 kV spray voltage, 15 sheath gas, and 5 auxiliary gas, operated in positive ion mode. Calibration was performed prior to analysis using the PierceTM Positive and Negative Ion Calibration Solutions (ThermoFisher Scientific). Acquired data were converted from .raw to .mzXML file format using RawConverter. To monitor possible technical variability, aliquots of each of the individual samples were combined to make technical replicates, which were run every 5 μl samples. Coefficients of variations (CV = standard deviation/mean) were calculated for each metabolite across tech mixes (generated by mixing 5 μl of all the samples being tested) and only metabolites with CVs <20% were considered for this study. In addition, in each experiment, several lysis solution aliquots were run as blanks for artifact identification. Metabolite assignments to KEGG compounds were performed using MAVEN (Princeton, NJ, USA) on the basis of accurate intact mass, high-resolution-based determination of chemical formulae, retention times against an in house standard library of ~1000 compounds, including two custom synthesized isomers for this study for both 3-OH and 5-OH-kynurenamine. Peak areas were exported for further statistical analysis.

#### 3-HKA identification

Chromatography was performed on a Thermo Ultimate 3000 High Performance Liquid Chromatography system (Thermo Scientific, San Jose, CA, USA). Analytes were separated on a Kinetex EVO C18 column (100 × 2.1 mm, 2.6 µm, Phenomenex, Torrance, CA, USA) connected with a guard column (2.1 × 5 mm, Phenomenex). LC eluent A was an aqueous solution containing 0.1% formic acid and eluent B was acetonitrile containing 0.1% formic acid (described in the “Reagents” section). The gradient was initiated with 30% eluent B and continued with linear increase to 70% B in 18 min. Then, the percentage of B increased to 100% in 2 min and this condition was maintained for 5 min. The system returned to 70% B in 1 min and it was re-equilibrated for 4 min (run time: 30 min). The column and autosampler temperatures were kept at 40 and 16 °C, respectively. The flow rate was 0.35 mL min^−1^ and injection volume 5 µL.

The mass spectrometer Q-Orbitrap Plus (Thermo Scientific) was equipped with heated electrospray ionization (HESI-II) source operating in positive ionization mode. The HESI-II temperature was set at 350 °C, the capillary temperature at 300 °C, the electrospray voltage at 3.50 kV. Sheath and auxiliary gas were set at 40 and 15 arbitrary units, respectively. The mass spectrometer analyser was controlled by the Xcalibur 3.0 software (ThermoFisher Scientific). The acquisition was achieved in t-SIM/dd-MS2 with a SIM isolation range of *m*/*z* 4.0. Data were acquired at a resolving power of 280,000 FWHM (*m*/*z* 200). Automatic gain control (AGC) was set at 1 × 10^6^ ion capacity with a maximum injection time (IT) of 600 ms. The precursor ion, filtered by the quadrupole (isolation width: *m*/*z* 2.0), was fragmented with stepped collision energies (CE) at 30, 50, and 100 eV. A resolving power of 175,000 FWHM (*m*/*z* 200) was applied for the measure of productions with AGC target and IT set at 5 × 10^5^ ions and 80 ms, respectively.

#### Quantitative analysis of 3-HKA

One hundred microliters of sample solution were added to 1.0 ml of acetonitrile. The suspension was vortexed, and then ultra-centrifuged at 9000 × *g* for 5 min at 5 °C. Five hundred microliters of the collected supernatant were incubated with 533 μl of a dansylchloride solution (1% (w/v) in acetone) (described in the “Reagents” section). The pH of the resulting solution was adjusted at a value of 8.0 by adding 0.1 M aqueous Na_2_CO_3_ solution. The mixture was heated for 60 min at 40 °C and then filtrated through a 0.45 µm nylon syringe filter. A solid residue was then obtained through rotatory evaporation. The dry residue was dissolved in 250 µl of acetonitrile and 100 µl of H_2_O and then analyzed by HPLC-DAD using the following conditions: column, Luna C18 (250 × 4.6 mm, 5 μm, 110 Å, from Phenomenex, cat# 00G-4041-E0); mobile phase; eluent A (0.1% formic acid in water) and eluent B (0.1% formic acid in acetonitrile), gradient program: 0–5 min, from 10 to 25% B; 5–10 min, from 25 to 40% B; 10–15 min from 40 to 55% B; 15–25 min, from 55 to 70% B; 25–35 min, from 70 to 85% B; 35–40 min, from 85 to 100% B; 40–60 min, the eluent is kept constant to 100% B; flow rate, 1.0 ml/min; column temperature, 20 °C; analysis was monitored at 254 nm. Each analysis was run after column equilibration with 10% B for 20 min at a 1.0 ml/min flow rate. A further 15 min column washing with a methanol/acetonitrile-50/50 (v/v) solution was always performed before column equilibration. The HPLC-DAD study was performed on a Waters ALLIANCE 2695 Separations Module system equipped with a quaternary, low-pressure mixing pump and in-line vacuum degassing, an autosampler with maximum capacity of 120 vials and a column heather/cooler. The system is endowed with a photodiode array detector (Waters 2996). The data management was made by means of Waters® Millennium®32 Software. The chemical identity of the dansylated biogenic amine was verified by high-resolution mass spectrometry (Orbitrap LC-MS Thermo Scientific) using dansylated 3-HKA as standard (MW 646.19 g/mol).

#### Immunohistochemistry

LECs were seeded on sterile 0.1% gelatin-coated coverslips in 35-mm petri dishes. Up to 70 to 80% confluence growth, cells were washed with PBS and fixed in 4% paraformaldehyde for 15 min at room temperature. After washing them three times in PBS, cells were permeabilized with 0.1% Triton X-100 (MILLIPORE-Sigma-Aldrich, cat# 9002-93-1) for 20 min and incubated in blocking solution (1X PBST contain 1% BSA, MILLIPORE-Sigma-Aldrich, cat# A7888-100g) for 1 h. Cells were then incubated with the following primary antibodies: AF488-conjugated anti-mouse Ido1 (Santa Cruz, cat# 53978, dilution 1:500) or (ENZO, cat# ALX-804-902-0100, dilution 1:200); Lyve 1 (R&D System, cat# AF2125, dilution 1:100); biotin-conjugated anti-mouse Podoplanin (Biolegend, cat# 127403, dilution 1:200), overnight at 4 °C. After washing thrice with 1X PBST, cells were incubated with the following secondary antibodies, Anti-Goat Alexa Fluor 405 (Abcam, cat# ab175664, dilution 1:100) and streptavidin AF 568 conjugate (Molecular Probes, cat# S11226, dilution 1:200) for 1 h at room temperature. Cells were imaged under the Zeiss fluorescence microscope.

Mesenteric lymphatic vessels and the thoracic duct were harvested from Prox1-cre-ERT2-tdTomato mice as previously reported^25^. Isolated vessels were perfused with 1XPBS (MILLIPORE-Sigma-Aldrich, cat# 11666789001), fixed for 1 h in 4%PFA, and washed three times for 15 min each with 1XPBS. Vessels were then permeabilized with 0.3% Triton x100 (MILLIPORE-Sigma-Aldrich, cat# 9002-93-1) for 30 min followed by three more washes in 1X PBS. The vessels were then blocked for 1 h in 5% bovine serum albumin (Sigma, cat# A7888-100g) in 0.05% Tween-20 (MILLIPORE-Sigma-Aldrich, cat# P9416) in 1X PBS (Millipore Sigma, cat# 11666789001) for 1 h at RT. The vessels were incubated overnight at 4 °C in 1X PBST containing primary antibody IDO1 (ENZO, cat# ALX-804-902-0100, dilution 1:200). After washing three times for 15 min each with 1XPBST, the vessels were incubated for 1 h in 1XPBST containing goat anti-mouse FITC (Southern Biotech, cat# 1070-02, dilution 1:500). After further washing in 1XPBST the vessels were mounted on a glass slide under #1.5 coverslips using ProLong Gold antifade reagent (Life Technologies, cat# P369345). The slides were imaged under LSM 880 with Airyscan confocal microscope.

C57BL/6 mice and Prox1-td-Tomato mice lymph nodes were harvested, fixed in 10% formalin (Fisher Scientific, cat# SF100-4) for 1 h, and included in OCT medium (Fisher Scientific, cat# 23-730-571). Six-micrometer tissue sections were boiled for 10 min in citric acid, pH6 (antigen retrieval) (MILLIPORE-Sigma-Aldrich, cat# C9999), washed three times with 1X PBS, and blocked with 3%BSA in 1XPBST for 1 h at RT followed by primary antibody incubation for AF488-conjugated IDO1 (ENZO, cat# ALX-804-902-0100, dilution 1:200) and anti-Lyve 1 (Angiobio, 11-034, dilution 1:100) for overnight at 4 °C. After washing three times for 15 min each with 1XPBST, samples were incubated with the secondary antibody, AF 647-conjugated goat anti-rabbit (Jackson Immunoresearch, Inc., cat# 111-606-144, dilution 1:500) for 1 h at RT. After further washing in 1X PBST, ProLong Gold antifade reagent (Life Technologies, catalog # P36941) was applied to each sample. The slides were imaged under LSM 880 with Airyscan confocal microscope.

#### Contact hypersensitivity-response in ear skin

Seven-eight-week-old C57BL/6 females were sensitized by applying topically 2% oxazolone (4-ethoxymethylene-2-phenyl-2-oxazoline-5-1, MILLIPORE-Sigma-Aldrich, cat# 862207) on shaved abdomen and paws. Five days later, mice were challenged by applying 1% oxazolone on the ears and 24 h later animals were sacrificed and ear skin tissue was harvested for FACS-sorting.

#### *Ido1* gene expression in blood and lymphatic endothelial cells

CHS-inflamed and steady-state ears from two animals per condition were pooled, cut into small pieces, and enzymatically digested in a 4 ml mixture of 10 mg/ml collagenase IV (Worthington Biochemical Corporation, cat# LS004186), 5 mg/ml dispase II (Roche, cat# 04942078001), 0.1 mg/ml DNase I (Millipore Sigma, cat# 260913-10MU), and 1 mM CaCl_2_ (Millipore Sigma, cat# 449709) in PBS at 37 °C for 15 min in rotation. After passaging suspensions through a 40 µm cell strainer (Fisher Scientific, cat# 08-771-1), resulting single-cell suspensions were stained at 4 °C with APC/Cy7 anti-mouse CD45, dilution 1:200 (BioLegend, cat# 103115), PE anti-mouse CD31 dilution 1:200 (BioLegend, cat# 102407), APC anti-mouse Podoplanin dilution 1:200 (BioLegend cat#127409), AF488 anti-mouse LYVE-1 dilution 1:100 (eBioscience, cat# 53-0443-82), and 7-AAD (BioLegend, cat# 420403). Different endothelial cell (EC) populations were isolated on a FACSAria Cell Sorter (70 mm nozzle, BD Biosciences) and sorted into PicoPure RNA extraction buffer (ThermoFisher Scientific, cat# KIT0204). RNA was extracted and genomic DNA was eliminated using the PicoPure RNA isolation kit (ThermoFisher Scientific, cat# KIT0204). Amplified cDNA was prepared using Ovation Pico WTA System V2 (NuGen) and quantitative PCR analyses of *Ido1* (primer sequence) and *Rpl0* (primer sequence) gene expression were performed on a Fast Real-time PCR system (ThermoFisher Scientific).

#### Cytokine quantification

Cell-free supernatant was analyzed using the human Cytokine Array Proteome Profiler antibody array system (R&D Systems: cat# ARY005B) according to the manufacturer’s instructions. Cytokines and chemokines, which showed statistically different up or downregulation following 3-HKA treatment, were further validated and quantified using the Bio-Plex pro human cytokine immunoassays (BioRad, cat# 12007283) according to the manufacturer’s instructions. The Bio-plex pro human cytokine standard group I was used as a standard for the assay. The concentrations of the cytokines were statistically analyzed using the Mann–Whitney test, and plotted with GraphPad Prism software package.

Skin and kidney total lysates from psoriasis and nephrotoxic mice models and control animals were analyzed using a mouse cytokine array kit (Quantibody^®^ Mouse Th17 Array 1, from RayBiotech Inc., Norcross, GA, cat# QAM-TH17-1), which detects 18 mouse Th1, Th2, and Th17 cytokines, using the manufacturer specifications. An Axon scanner 4000B with GenePix software was used to collect fluorescence intensities. The QAM-TH17-1 Q-Analyzer v8.18.4 excel software was further employed to analyze the final extracted data using the median fluorescence intensities corrected for the background, as recommended by the manufacturer. Individual calibration curves for each cytokine (fitted to linear or log–log regression functions) were adjusted for obtaining the best fit (*R*^2^ > 0.98) by removing data corresponding to saturation of the fluorescence signal. The final cytokine concentrations (pg/ml) were interpolated from the calibration curves and plotted as average ± standard deviation. The statistical significance was determined using the Holm–Sidak method, with alpha = 0.05 by applying multiple *t*-tests analysis where each row (corresponding to individual cytokine data) was analyzed individually, without assuming a consistent SD in GraphPad Prism 8.1.1. An independent analysis for statistics significance was performed using one-way or two-way ANOVA and followed by Tukey’s multiple comparison test when appropriate.

### RT-PCR

Real-time PCR analysis was carried out on mRNA extracted from untreated or IFN-γ-treated LEC using specific primers (Supplementary Data 3). Data were calculated as the ratio of gene to Gapdh expression by the relative quantification method (ΔΔCT; average ± SD of triplicate determination). Values are reported as normalized transcript expression in the samples treated with IFN-γ relative to normalized transcript expression in control cultures (in which fold change = 1; dotted line).

### Skin test assay

A skin test assay was used for measurements of major histocompatibility complex class I–restricted delayed-type hypersensitivity (DTH) responses to the HY peptide (i.e., male minor antigen; sequence WMHHNMDLI) in C57BL/6 female recipient mice, as described^11,12,26^. For in vivo immunization, 3 × 10^5^ peptide-loaded CD8^−^CD11c^+^ splenic DCs, combined with a minority fraction (5%) of the same DCs, either untreated or pretreated with 3-HKA at 1 µM for 24 h, were injected i.v. into recipient mice. Two weeks later, a DTH response was measured to intrafootpad challenge with the eliciting peptide, and results were expressed as the increase in footpad weight of peptide-injected footpads over that of vehicle-injected (internal control) counterparts.

### Imiquimod-induced psoriasis

C57BL/6 mice were randomly divided into four groups. Group I: untreated control; Group II: imiquimod; Group III: 3-HKA; Group IV: Imiquimod+ 3-HKA (50 mg/kg). All mice were shaved on the dorsal skin (1 cm^2^ area). The imiquimod cream (5% Imiquimod; Aldara, TARO Pharmaceuticals, cat# 333028) was topically administered on the mouse back every day for 6 consecutive days for groups II and VI. Control mice were treated similarly with control vehicle cream (VWR; cat# 56614-414). 3-HKA (50 mg/kg of body weight) was administered via ip injection once a day for 6 consecutive days in groups III and IV. Mice were weighed daily and monitor for the erythema, scaling, and induration for the severity of psoriasis. Mice were sacrificed on day 7 by ketamine/xylazine injection followed by cervical dislocation.

The dorsal skin tissue was excised from the mouse after sacrifice. All tissues were fixed in 10% neutral buffered formalin for 24 h and then transferred to 70% ethanol. After a series of ethanol gradient dehydration and xylene cleaning, tissues were embedded in paraffin and sectioned at 5 µm for histochemical staining and immunohistochemistry staining. Hematoxylin and eosin stain (H&E) was performed in an automated stainer (Leica Autostainer XL) following routine procedures. Histomorphology of each H&E slide was evaluated by a board-certified pathologist, at three randomly chosen regions and a separate lesion score was provided for each region. Features that were assessed were adapted from Baker et al.^27,28^ and included those of the keratin layer (orthokeratosis, parakeratosis), epidermis (hyperplasia, formation of rete ridges, ulceration), and the dermis (lymphocytic infiltration, superficial congestion, hemorrhage, necrosis). The severity of each finding was scored blindly semi-quantitatively as follows: 0 = no lesion; 1 = minimal; 2 = mild; 3 = moderate; 4 = severe. Inflammatory infiltrates were semi-quantified at high-power field on the H&E slides using a multi-tier system as following: 0 for no inflammation or rare inflammatory cells within normal limit, 1 for total inflammatory cell clusters 1–3 foci, 2 for total inflammatory cells clusters 4–10 foci, 3 for total inflammatory cells clusters >10 foci. The stained slides were scanned with the OptraSCAN OS15 system. The scanned files were analyzed using Aperio Imagescope for the number, intensity, and area of defined positive pixels. The images were captured with Olympus cellSens Entry software at indicated magnifications.

### Nephrotoxic nephritis

Nephrotoxic serum nephritis was induced as follows^29^: ten-week-old female 129sv/J mice were immunized with rabbit IgG emulsified with CFA by intraperitoneal injection on day 0. Starting at day +4, mice received daily intraperitoneal injections of 50 mg/kg of 3-HKA (*n* = 6) or vehicle control (*n* = 8) until sacrifice. On day 5, mice were intravenously injected with either nephrotoxic serum (N-Ab) or PBS. Starting on day 7, mice were monitored daily for proteinuria via uristix (Siemens Healthcare Diagnostic, cat# 10312569). Terminal serum levels of blood urea nitrogen (BUN) were measured using quantichrom urea assay kit (BioAssay System, cat# DIUR-100) according to manufacturer’s instructions. To assess model induction, mouse anti-rabbit IgG and rabbit anti-mouse GBM levels were measured^29^.

### Flow cytometry staining and analysis of lymph node cells

Lymph nodes were harvested from untreated, imiquimod, or imiquimod and 3-HKA treated C57BL/6J mice either with or without tamoxifen, as indicated, and processed to obtain a single-cell suspension for antibody staining and flow cytometry analysis. Briefly, lymph nodes were pulled apart into small pieces using sharp forceps and incubated at 37 °C for 30 min in RPMI media (Corning, cat# 15-040-cv) containing 400 U/ml of collagenase D (Roche, cat# 11088858001). Lymph nodes were pressed against a 70 µm filter (Fisher Scientific, cat# 08-771-2) to obtain a single-cell suspension, washed with RPMI media containing 10% fetal bovine serum (Sigma, cat# F0926) and resuspended in cold FACS buffer (PBS, 1% BSA, 0.1% sodium azide) in preparation for antibody staining. Cells were incubated with the 2.4G2 antibody at 4 °C for 15 min, followed by staining with indicated antibodies at 4 °C for 30 min. For staining of intracellular markers and transcription factors, cells were fixed and permeabilized after the surface stain incubation using the FoxP3/Transcription factor stain kit (eBioscience, cat# 00-5523-00) following the manufacturer’s instructions. Stained cells were analyzed on the BD Aria III cytometer and data processed using FlowJo v10.5.3 software (BD).

### Generation of LEC-IDO1-deleted mice

As part of the EUCOMM Consortium mouse targeting facility, L1L2_Bact_P cassette was inserted at position 24592161 of Chromosome 8 upstream of the critical exon(s) (Build GRCm38). The cassette is composed of an FRT site followed by lacZ sequence and a loxP site. This first loxP site is followed by a neomycin resistance gene under the control of the human β-actin promoter, SV40 polyA, a second FRT site, and a second loxP site. A third loxP site is inserted downstream of the targeted exon(s) at position 24591248. Mice were crossed for three generations to C57BL6/J. A conditional allele was created by crossing to Flp recombinase expression (C57BL/6J Flpe) in mice carrying this allele. Genome removal of LacZ and Neo was confirmed by PCR in offspring, Flpe was removed by additional crosses to C57BL/6J, after which the line was made homozygous for Ido1 floxed (indoleamine 2,3-dioxygenase 1; targeted mutation 1a, Wellcome Trust Sanger Institute, Cambridge, UK. MGI ID: 4432044 EM:05803). These mice were crossed to Prox1-cre-ERT2-Rosa26-TdT (http://www.informatics.jax.org: 007909 and 022075 and generous gift of G. Randolph; Washington University) to achieve LEC-specific deletion of *Ido1*. Each mouse was genotyped from ear biopsies using RT-PCR with specific primers and probes designed for each gene. To genotype the mice the following sets of primers/probes set were used: L1L2-Bact-P MD and Ido1-4 KO (to detect LoxP-IDO1); Ido1-4 WT (to detect the presence of the wild-type IDO1 gene); Prox1-3 KO and CRE (to detect Prox1-cre-ERT2); ROSA26 WT (to detect the wild-type ROSA26 locus); tdRFP (to detect tDTomato in the Rosa26 locus); and L1L2-Bact-P EX (to detect Cre-Lox recombination).

### Statistical analysis

Statistical analysis was performed using Windows GraphPad Prism 8 (GraphPad Software, La Jolla, CA). Numerical results are reported as mean ± SE or ±SDV when appropriate. Data are derived from a minimum of three independent experiments unless stated otherwise. Comparisons of the expression of human proteins (STAT1, STAT2, NF-κΒ, cytokines, and chemokines) were performed using the two-tailed paired parametric *t* test. A comparison between more than two groups was performed using two-tailed unpaired one-way analysis of variance (ANOVA). Results were considered statistically significant if *p* ≤ 0.05. Independent multiple *t*-tests discovery analysis was performed in some experiments aimed to compare the changes in the protein expression profiles across many proteins within one pathway, such as across NF-κΒ or STAT pathways. In such cases, the significance was determined using the two-stage linear step-up procedure of Benjamini, Krieger, and Yekutieli, with a false discovery rate *Q* = 3%. Each data set corresponding to one targeted protein was analyzed individually, without assuming a consistent SD. In the case of the cytokines arrays the statistical significance was determined using the Holm–Sidak method, with alpha = 0.05 by applying multiple *t*-tests analysis where each row (corresponding to targeted protein cytokine data) was analyzed individually, without assuming a consistent SD. An independent analysis of statistical significance for the cytokine arrays employed the two-stage linear step-up procedure of Benjamini, Krieger, and Yekutieli, with a false discovery rate *Q* = 1–3% as described above.

### Reporting summary

Further information on research design is available in the Nature Research Reporting Summary linked to this article.

## Supplementary information

Supplementary Information

Peer Review File

Descriptions of Additional Supplementary Files

Supplementary Data 1

Supplementary Data 2

Supplementary Data 3

Reporting Summary

## Data Availability

Proteomic data have been deposited to the ProteomeXchange Consortium via the PRIDE partner repository with the accession code PXD015865. All other data are present in the article and its Supplementary Information files or from the corresponding author upon reasonable request. Source data are provided with this paper.

## Source data

Source Data
