# Supplementary material for: 3-hydroxy-L-kynurenamine is an immunomodulatory biogenic amine
Source: Nat Commun. 2021 Jul 21;12:4447. doi: 10.1038/s41467-021-24785-3 (PMC8295276; doi:10.1038/s41467-021-24785-3)
Supplement: Supplementary file 1 — Supplementary Information [file 41467_2021_24785_MOESM1_ESM.pdf]

Supplementary Informations

3-hydroxy-L--kynurenamine is a novel immunomodulatory biogenic amine

[1]Cristina C. Clement, [2]Angelo D'Alessandro, [1]Sangeetha Thangaswamy, [3]Samantha Chalmers, [3]Raquel Furtado, [1]Sheila Spada, [4]Giada Mondanelli, [5]Federica Ianni, [2]Sarah Gehrke, [4]Marco Gargaro, [4]Giorgia Manni, [6]Luisa Carlota Lopez Cara [7]Peter Runge, [8]Tsai Wanxia Li, [9]Sinem Karaman, [7]Jorge Arasa, [10]Ruben Fernandez-Rodriguez, [11]Amanda Beck, [5]Antonio Macchiarulo, [8]Massimo Gadina [7]Cornelia Halin, [4]Francesca Fallarino, [10]Mihaela Skobe, [12]Marc Veldhoen, [13]Simone Moretti, [1,14]Silvia Formenti, [1,14]Sandra Demaria, [15]Rajesh K. Soni, [13]Roberta Galarini, [5]Roccaldo Sardella, [3]Gregoire Lauvau, [3,3a,3b]Chaim Putterman, [9]Kari Alitalo [4,11*]Ursula Grohmann, [1,11,14,16*]Laura Santambrogio

[1]Department of Radiation Oncology, Weill Cornell Medicine, 1300 York Ave, New York, NY, 10065, USA. [2]Department of Biochemistry and Molecular Genetics, University of Colorado Denver, Anschutz Medical Campus, 12801 E. 17th Ave., Aurora, CO, 80045, USA. [3]Division of Rheumatology and the Department of Microbiology and Immunology, Albert Einstein College of Medicine, 1300 Morris Park Avenue, New York, NY 10461, USA. [3a]Azrieli Faculty of Medicine, Bar-Ilan University, Zefat, Israel 13100. [3b]Research Institute, Galilee Medical Center, Nahariya, Israel 22001. [4]Department of Medicine and

Surgery, University of Perugia, 1 P.le Gambuli, Perugia 06132, Italy. [5] Department of Pharmaceutical Sciences, University of Perugia, Via del Liceo 1, Perugia 06123, Italy [6] Department of Pharmaceutical & Organic Chemistry, Faculty of Pharmacy, University of Granada, 18010 Granada, Spain. [7]Institute of Pharmaceutical Sciences, ETH Zurich, Vladimir-Prelog-Weg 4, Zurich, 8093, Switzerland. [8] Translational Immunology Section, National Institute of Arthritis Musculoskeletal and Skin Diseases, NIH Bldg. 10, Room 10C211, 10 Center Drive, Bethesda, MD, 20892, USA. [9] Wihuri Research Institute and Translational Cancer Medicine Research Program, University of Helsinki, Haartmaninkatu 8, (Biomedicum 1, A515b), 00290 Helsinki, Finland. [10]Department of Oncological Sciences and Tisch Cancer Institute, Icahn School of Medicine at Mount Sinai, New York, NY, 10029, USA. [11]Department of Pathology, Albert Einstein College of Medicine, 1300 Morris Park Avenue, New York, NY 10461, USA (U.G. as Visiting Professor). [12] Instituto de Medicina Molecular | João Lobo Antunes, Faculdade de Medicina da Universidade de Lisboa, Av. Professor Egas Moniz, Lisbon, 1649-028, Portugal. [13]Istituto Zooprofilattico Sperimentale dell'Umbria e delle Marche "Togo Rosati, Via G. Salvemini, 1, Perugia 06126, Italy. [14]Sandra and Edward Meyer Cancer Center, 1300 York Avenue, New York, NY, 10065 USA. [15]Proteomics and Macromolecular Crystallography Shared Resource, Herbert Irving Comprehensive Cancer Center, Columbia University Irving Medical Center, New York, NY; [16]Caryl and Israel Englander Institute for Precision Medicine of Weill Cornell Medicine, 413 E 69th Street, New York, NY, 10065, USA.

[*]Co-Corresponding Authors:

las4011 @med.cornell.edu (LS) and ursula.grohmann@unipg.it (UG)

Supplementary Figures

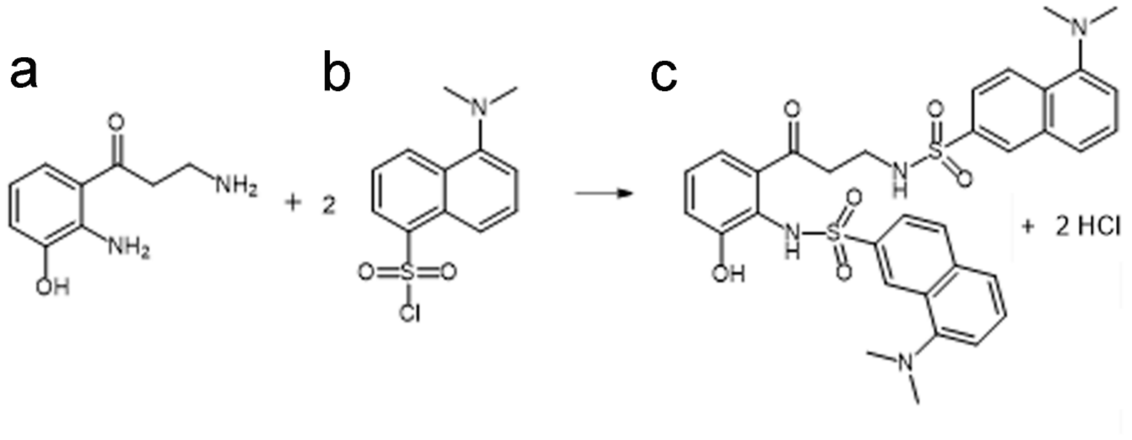

Supplementary Figure 1

Supplementary Figure 1: Schematic of 3-HKA dansylation

a,b,c) 3-Hydroxykynurenamine was reacted with dansyl-chloride (b) producing the di-dansylated 3-hydroxykynurenamine derivative (c). The stable derivatization product was quantitatively analysed by HPLC-DAD (**Figure 1 b**).

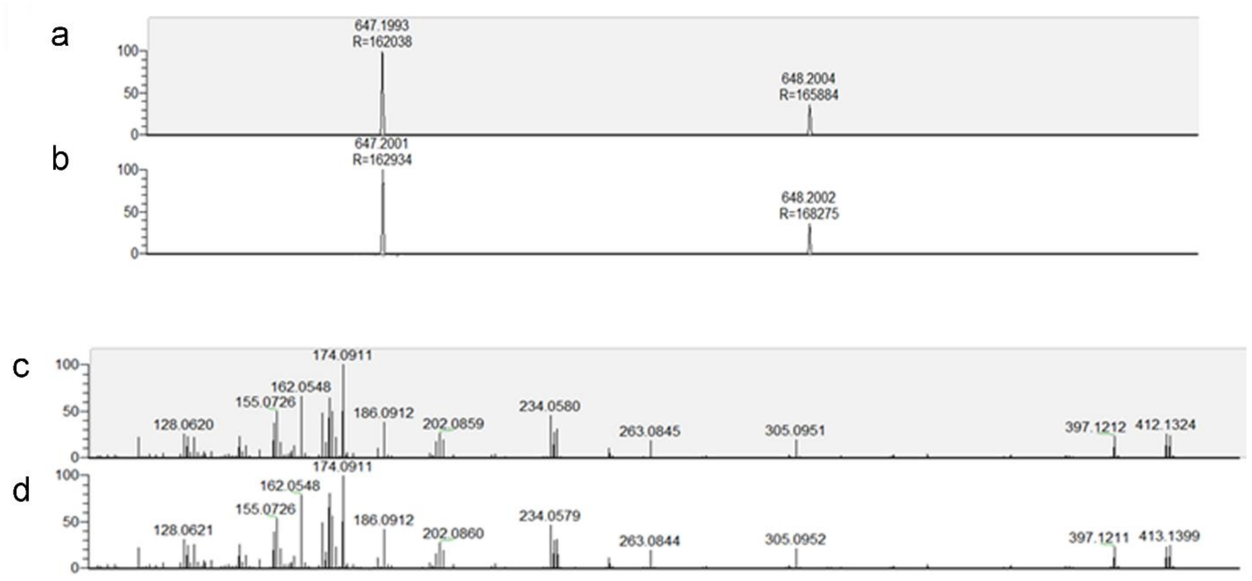

Supplementary Figure 2

Supplementary Figure 2: Quantification of dansylated-3-HKA

To detect and quantify 3-HKA in biological fluids, the biogenic amine was dansylated as reported in Supplement Figure 1.**a,b)** To further verify the 3-HKA identity an additional approach (LC-HRMS/MS) was utilized. The protonated molecule of the reference di-dansylated 3-hydroxykynurenamine standard (theoretical *m/z* 647.1993) (**a**) was compared with a dansylated blood sample from control mice (**b**), within a 2 ppm mass error. **c,d)** Additionaly, at least six fragment ions (accurate *m/z* 412.1324, *m/z* 397.1212, *m/z* 305.0951, *m/z* 263.0845, m/z 234.0580 and *m/z* 162.0548) in the MS^2 spectrum of the reference di-dansylated 3-hydroxykynurenamine standard (**c**) supported 3-HKA identity in the dansylated blood sample from control mice (**d**).

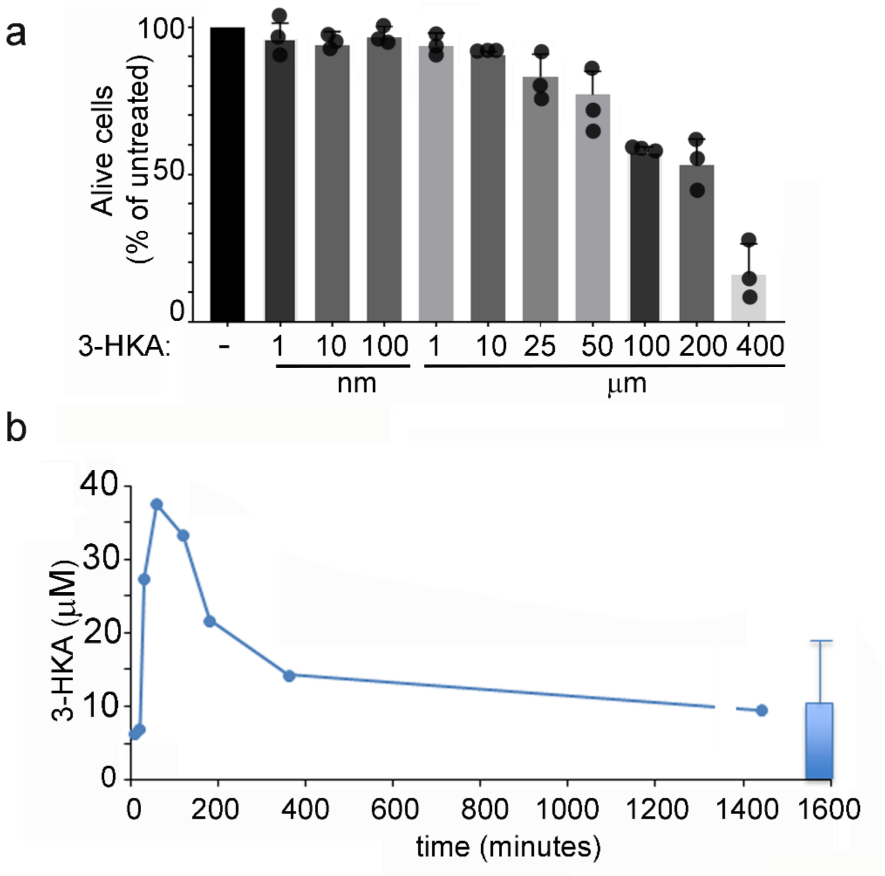

Supplementary Figure 3

Supplementary Figure 3: MTT assay to determine dosage of 3-HKA toxicity and half-life a) Human monocyte-derived DCs were cultured for 24 hours with titrated amount of 3-HKA. Cytotoxicity was analyzed using the MTT colorimetric assay (Abcam), which assess the number of viable cells. Data are reported as average ± SD of viable cells in biological triplicates (n=3). **b)** To evaluate the potential use of 3-HKA in animal models of inflammatory condition, we first evaluated its pharmacokinetic properties. 3-HKA was labeled via a dansylating procedure and injected i.p. into C57BL6/J mice (at the dose of 50 mg/kg). Sera were collected at different time points for HPLC/MS quantification. Injected 3-HKA reached its C_{max} around 1.5 hours, was still detectable in sera at ~24 hours after administration, and presented a $t_{1/2}$ of 257 minutes, considerably higher than that of other Trp metabolites such as kynurenines (i.e., ≤~30 minutes)[33]. Sera levels of 3-HKA following *in vivo* i.p administration (50mg/Kg) administered in C57BL/6J mice. Data

are reported as average ± SD of technical triplicates. n=2 biological duplicate. 3-HKA quantitation at each time point was calculated using 3HKA external standard.

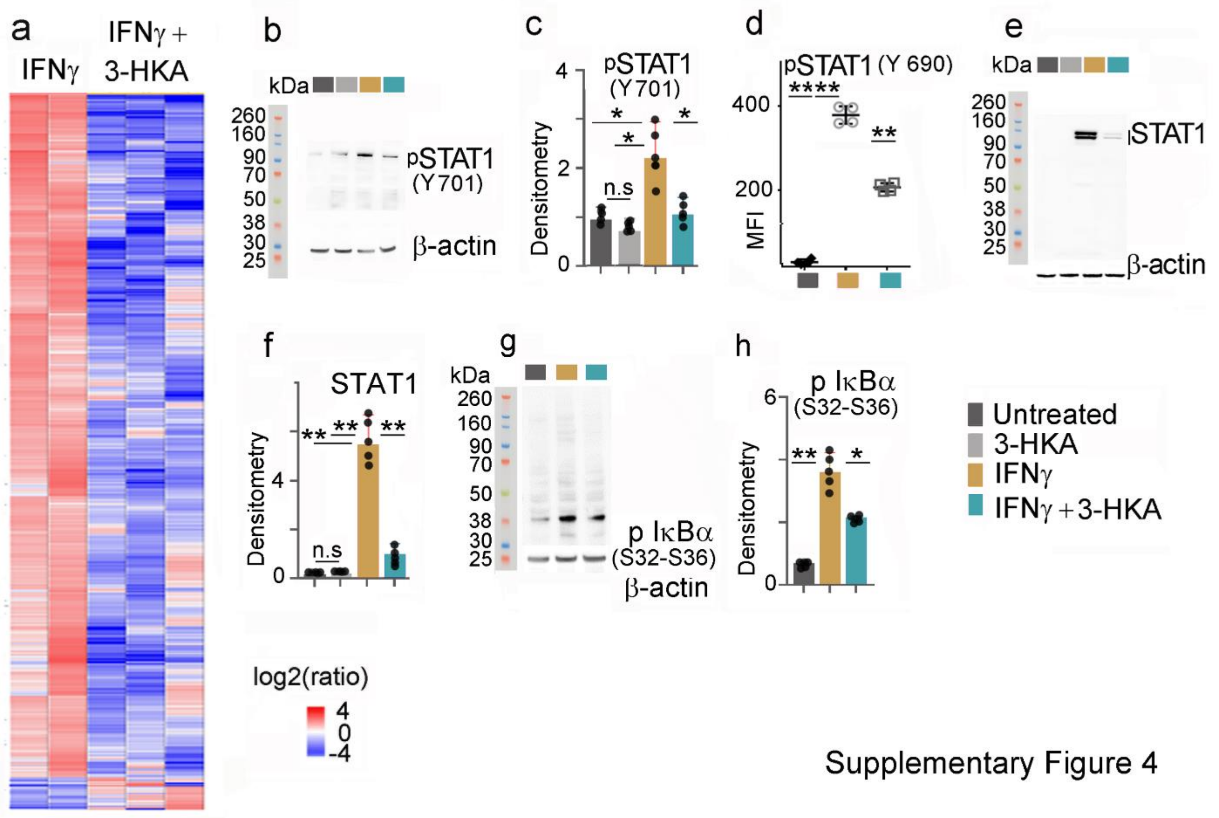

Supplementary Figure 4: 3-HKA inhibits IFN-γ-mediated NF-κB activation in DCs

a) Protein profile heat map of mouse splenic DC proteome from cells treated with 50 ng/ml IFN-γ for 24 hr in presence or absence of 1 μm 3-HKA. The heat map shows the Log2 (ratios) of the average protein abundances generated by the PEAKS Q module implemented in PEAKS 8.0/8.5 using the label free quantitative (LFQ) MS1 analysis from biological replicates (n=2 for IFN-γ-treated DCs and n=3 for the IFNγ+3-HKA treatments). The proteomic data are available at ProteomeXchange Consortium via the PRIDE partner repository with the dataset identifier PXD015865. **b)** Representative Western blot analysis of phosphorylated STAT1(Y701) from human monocyte-derived DCs treated or untreated with 1 μM 3-HKA with or without 50 ng/ml IFN-γ for 20 min. **c)** Densitometric analysis of phosphorylated STAT1(Y701), normalized to β-actin expression. Data from biological replicates (n=5) are plotted as mean relative expression ± SD. Significance levels are reported as $p<0.05$ (*), $p<0.01$ (**) and $p<0.001$ (***) (two-way ANOVA followed

by Tukey's multiple comparison test). **d)** MFI for pSTAT1 analyzed in cell lysates, prepared as in (**b**) using the STAT Milliplex Luminex Assay. Data from biological replicates (n=4) are plotted as mean relative expression ± SD. Significance levels are reported as $p<0.05$ (*), $p<0.01$ (**) and $p<0.001$ (***) (two-way ANOVA followed by Tukey's multiple comparison test). **e)** Representative Western blot analysis of STAT1 from human monocyte-derived DCs treated or untreated with 1 µM 3-HKA after stimulation with or without 50 ng/ml IFN-γ for 24 hr. **f)** Densitometric analysis of STAT1, normalized to β-actin expression. Data from biological replicates (n=5) plotted as mean relative expression ± SD. Significance levels are reported as $p<0.05$ (*), $p<0.01$ (**) and $p<0.001$ (***) (two-way ANOVA followed by Tukey's multiple comparison test). **g**) Representative Western blot analysis of STAT1 and the NF-κB pathway-associated proteins p65, p50 from cells treated as in (**e**). **h)** Densitometric analysis of pIκBα, normalized to β-actin expression. Data from biological replicates (n=5) plotted as mean relative expression ± SD. Significance levels are reported as $p<0.05$ (*), $p<0.01$ (**)$p<0.001$ (***) (two-way ANOVA followed by Tukey's multiple comparison test).

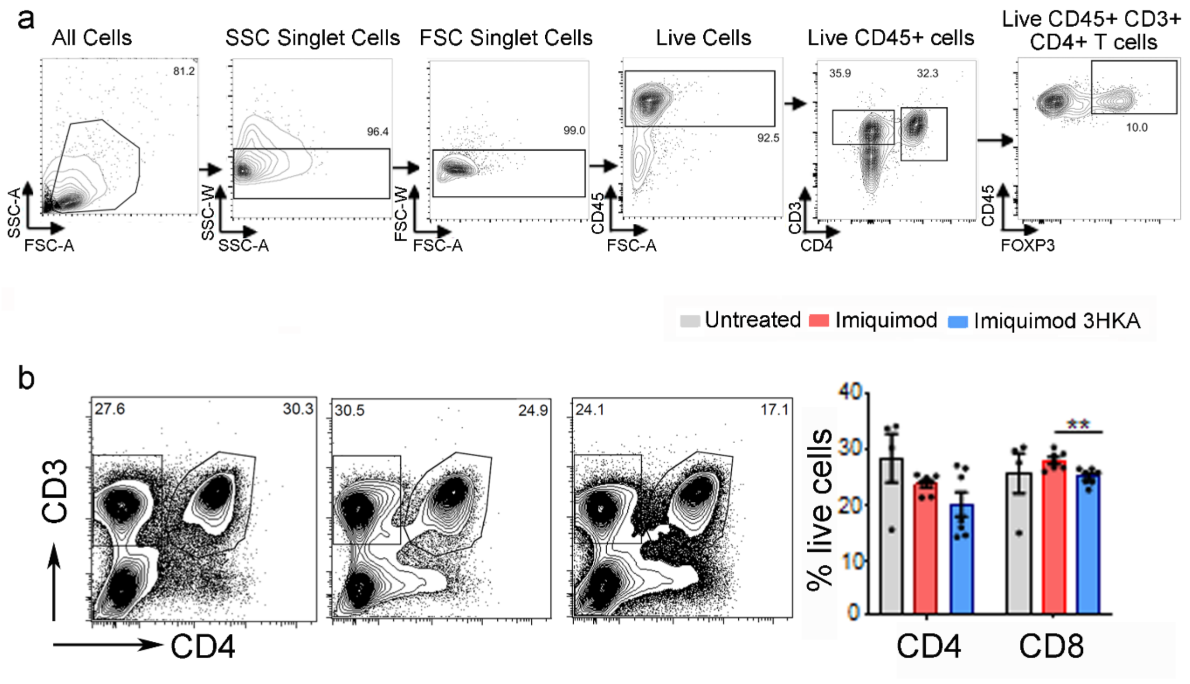

Supplementary Figure 5

Supplementary Figure 5: Reduced CD8^{+} T cells activation in psoriatic mice treated with 3-HKA.

a) Flow cytometry gating strategy on a representative lymph node sample for the indicated populations of live cells. **b)** Flow cytometry analysis on cells from the lymph nodes of untreated, imiquimod or imiquimod and 3HKA treated C57Bl/6 mice. Untreated (n=4 biologically independent samples), imiquimod (n=7 biologically independent samples) or 3-HKA plus Imiquimod treated C57BL/6J mice (n=7 biologically independent samples). Representative flow cytometry plots of indicated gated populations and summary frequencies of CD4^{+} and CD8^{+} T cells among live cells. Data are reported as average ± SEM analyzed by two-tailed unpaired student's-t test, conducted between indicated groups. Frequency of CD8+ T cells of live cells, imiquimod vs imiquimod 3HKA **p=0.0045.

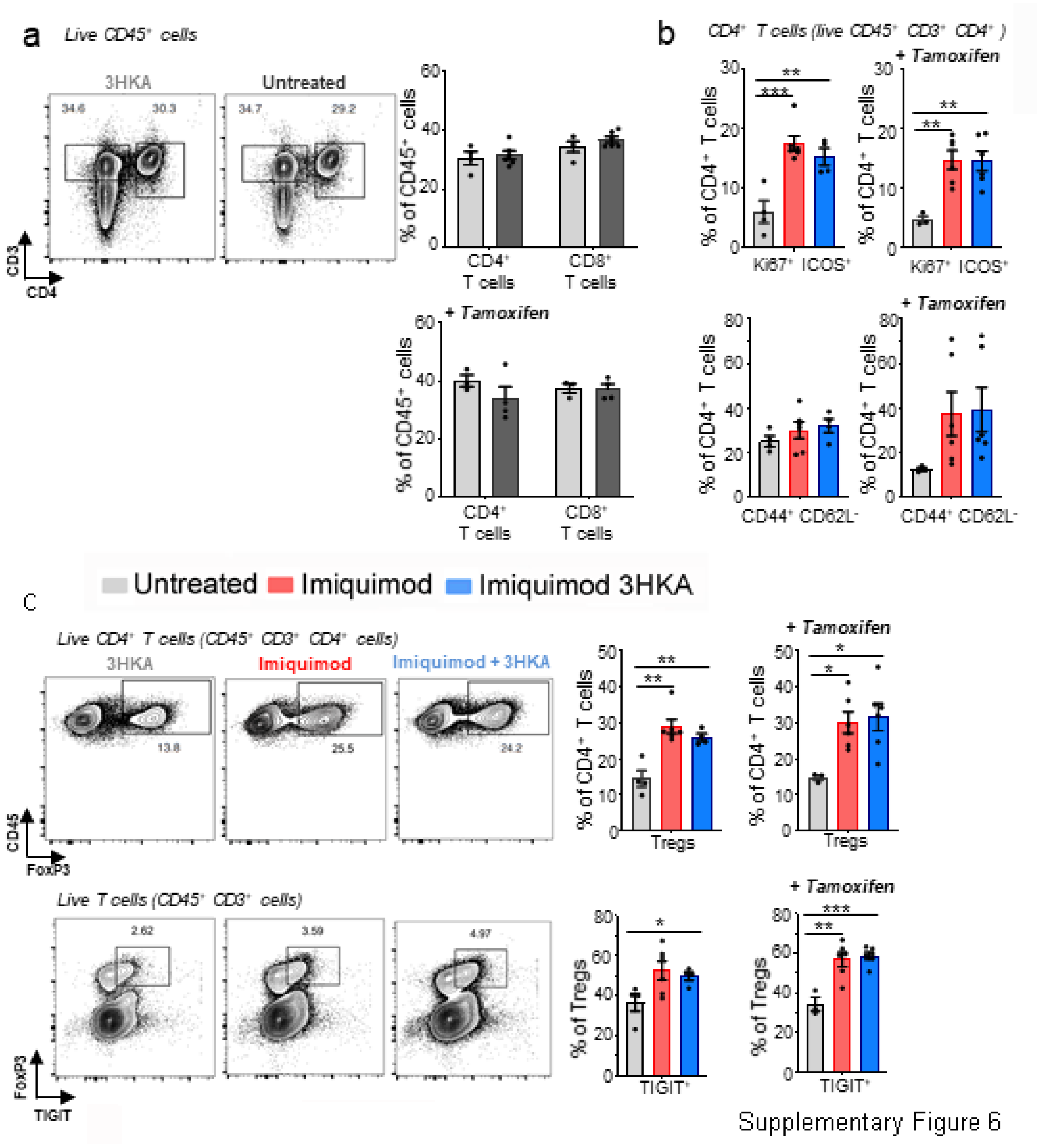

Supplementary Figure 6. CD4+ T cell subsets and Tregs in IDO-LEC KO mice. Flow cytometry analysis on cells from the lymph nodes of untreated, imiquimod or imiquimod and 3HKA IDO1-LEC KO mice with or without tamoxifen administration. Mice with tamoxifen administration are denoted as +Tamoxifen. Representative flow cytometry

plots of indicated gated populations and summary frequencies of **a)** $CD4^+$ and $CD8^+$ T cells ($CD3^+$) among $CD45^+$ cells. Untreated n=6 and 3HKA n=4. Data are presented as average ± SEM analyzed by two-tailed unpaired student's-t test. **b)** proliferating and activated subsets of $CD4^+$ T cells. Frequency of $Ki67^+$ $ICOS^+$ of $CD4^+$ T cells (3HKA vs. imiquimod - ***p=0.00089, 3HKA vs. imiquimoid 3HKA - **p=0.0082, imiquimod vs imiquimod 3HKA – ns p=0.294) and with tamoxifen (3HKA vs. imiquimod - ***p=0.00376, 3HKA vs. imiquimoid 3HKA - **p= 0.0055, imiquimod vs imiquimod 3HKA – ns p=0.931). Frequency of $CD44^+$ $CD62L^-$ of $CD4^+$ T cells (3HKA vs. imiquimod – ns p=0.354, 3HKA vs. imiquimoid 3HKA – ns p=0.118, imiquimod vs imiquimod 3HKA – ns p=0.714) and with tamoxifen (3HKA vs. imiquimod - *p=0.0363, 3HKA vs. imiquimoid 3HKA - **p=0.0031, imiquimod vs imiquimod 3HKA – ns p=0.944). Data are presented as average ± SEM analyzed by two-tailed unpaired student's-t test. **c)** frequency of $FoxP3^+$ (Tregs) cells among $CD4^+$ T cells (top panel, 3HKA vs. imiquimod - **p=0.0015, 3HKA vs. imiquimoid 3HKA - **p=0.00397, imiquimod vs imiquimod 3HKA – ns p=0.281 and with tamoxifen, 3HKA vs. imiquimod - *p=0.0104, 3HKA vs. imiquimoid 3HKA - *p=0.0191, imiquimod vs imiquimod 3HKA – ns p=0.745) and frequency of $TIGIT^+$ subsets among Tregs (bottom panel, 3HKA vs. imiquimod – ns p=0.0514, 3HKA vs. imiquimoid 3HKA - *p=0.0392, imiquimod vs imiquimod 3HKA – ns p=0.668, and with tamoxifen 3HKA vs. imiquimod - **p=0.0056, 3HKA vs. imiquimoid 3HKA - ***p=0.000241, imiquimod vs imiquimod 3HKA – ns p=0.664). (b-c) 3HKA n=4, Imiquimod n=6, Imiquimod + 3HKA n=4 mice in groups with and without tamoxifen administration. Data are presented as average ± SEM analyzed by two-tailed unpaired student's-t test conducted between indicated groups and significant p-values reported as *p<0.05, **p<0.01 and ***p<0.001.
