# Supplementary material for: 3-hydroxy-L-kynurenamine is an immunomodulatory biogenic amine
Source: Nat Commun. 2021 Jul 21;12:4447. doi: 10.1038/s41467-021-24785-3 (PMC8295276; doi:10.1038/s41467-021-24785-3)
Supplement: Supplementary file 3 — Descriptions of Additional Supplementary Files [file 41467_2021_24785_MOESM3_ESM.pdf]

Descriptions of Additional Supplementary Files

Supplementary Data 1
Description: Lymphatic Endothelial Cells proteomic analysis**.**

Supplementary Data 2
Description: Dendritic Cells proteomic analysis.

Supplementary Data 3
Description: PCR Primers.
