# Supplementary material for: 3-hydroxy-L-kynurenamine is an immunomodulatory biogenic amine
Source: Nat Commun. 2021 Jul 21;12:4447. doi: 10.1038/s41467-021-24785-3 (PMC8295276; doi:10.1038/s41467-021-24785-3)
Supplement: Supplementary file 7 — Reporting Summary [file 41467_2021_24785_MOESM7_ESM.pdf]

nature research

Corresponding author(s): Laura Santambrogio and Ursula Grohmann

Last updated by author(s): 5/27/2021

Reporting Summary

Nature Research wishes to improve the reproducibility of the work that we publish. This form provides structure for consistency and transparency in reporting. For further information on Nature Research policies, see Authors & Referees and the Editorial Policy Checklist .

Statistics

For all statistical analyses, confirm that the following items are present in the figure legend, table legend, main text, or Methods section.

n/a	Confirmed	
☐	☒	The exact sample size (*n*) for each experimental group/condition, given as a discrete number and unit of measurement
☐	☒	A statement on whether measurements were taken from distinct samples or whether the same sample was measured repeatedly
☐	☒	The statistical test(s) used AND whether they are one- or two-sided<br>*Only common tests should be described solely by name; describe more complex techniques in the Methods section.*
☒	☐	A description of all covariates tested
☐	☒	A description of any assumptions or corrections, such as tests of normality and adjustment for multiple comparisons
☐	☒	A full description of the statistical parameters including central tendency (e.g. means) or other basic estimates (e.g. regression coefficient) AND variation (e.g. standard deviation) or associated estimates of uncertainty (e.g. confidence intervals)
☐	☒	For null hypothesis testing, the test statistic (e.g. *F*, *t*, *r*) with confidence intervals, effect sizes, degrees of freedom and *P* value noted<br>*Give P values as exact values whenever suitable.*
☒	☐	For Bayesian analysis, information on the choice of priors and Markov chain Monte Carlo settings
☐	☒	For hierarchical and complex designs, identification of the appropriate level for tests and full reporting of outcomes
X	☐	Estimates of effect sizes (e.g. Cohen's *d*, Pearson's *r*), indicating how they were calculated

Our web collection on statistics for biologists contains articles on many of the points above.

Software and code

Policy information about availability of computer code

Data collection

2D-DIGE expression profiling of whole protein extracts from LEC : Gel images were acquired on a Typhoon 9400 scanner (GE Healthcare) and analyzed using DeCyder Software (V6.0, GE Healthcare). Proteins with an untreated/treated ratio of ± 2.5 were excised for MS/MS analysis. The 2D-DIGE experiments were performed and analyzed at Applied Biomics (http://www.appliedbiomics.com/).
nanoLC MS/MS: technical triplicates of the peptides mixtures were analyzed on a Q Exactive HF quadrupole orbitrap mass spectrometer (Thermo Fisher Scientific, Waltham, MA, USA). Metabolomic data were collected Vanquish UHPLC system coupled online to a high-resolution Q Exactive mass spectrometer (Thermo Fisher, Bremen, Germany) Statistical and data analysis was performed using GraphPad

Data analysis

Protein identification: In the case of the mouse LEC and primary DCs, the raw files from each technical and biological replicate were filtered, de novo sequenced and assigned with a protein ID using Peaks 8.0/8.5 software (Bioinformatics Solutions, Waterloo, Canada), by searching against the mouse (Mus musculus) Swiss-Prot database (March 2018; 91,343 entries). An independent validation of the MS/MS-based peptides and protein identification was performed with the Scaffold (version Scaffold_4.6.2, Proteome Software Inc.) using the compatible ".mzid" files exported from PEAKS 8.0/8.5. Data were validated using the false discovery rate (FDR) method built in PEAKS 8.0/8.5 and protein identifications were accepted if they could be characterized with a confidence score of (−10lgP) 15 and above for peptides and (−10lgP) 15 and above for proteins. Searches for protein ID were retrieved using a minimum of 1 peptide per protein, after data were filtered for less than 1.5% FDR for peptides and less than 1.5 % FDR for proteins identifications (p<0.05). The Scaffold built in option "MuDPIT" was used to combine multiple files from biological and/or technical replicates. Gene ontology (GO) annotations and analysis of networks and cellular pathways
Comparison of networks, functional analyses, biochemical and cellular pathways associated with the changes in the protein expression profiles in the LEC and mouse primary DCs in response to each treatment were generated by employing the ingenuity pathway analysis (IPA; Ingenuity Systems, Redwood City, CA, USA). For network generation, datasets containing gene identifiers (gene symbols) were uploaded into the IPA application together with their rescaled log2 transformation of protein's area ratios, extracted from label free quantitative (LFQ) MS1 analysis provided by the PEAKS Q module implemented in PEAKS 8.0/8.5.
The probability of having a relationship between each IPA indexed biological function and the experimentally determined genes was calculated by the right-tailed built-in Fisher's exact test. The level of significance was set to a P-value of <0.05. Accordingly, the IPA analysis identified the molecular and cellular pathways from the IPA knowledge library of established pathways that were most significant to the dataset (-log (p value)>2.0). For the quantitative analysis of the expression profiles, IPA assigned the "z-score" function to all

eligible canonical and cellular pathways (where a "z<-2 represent significant down-regulation while a z>2.0 represent a significant up-regulation of the selected pathway).

In the case of 2D-DIGE, the protein expression fold exported from DeCyder Software was used to perform the quantitative pathway analysis.

The mass spectrometry proteomics data have been deposited to the ProteomeXchange Consortium via the PRIDE partner repository with the dataset identifier PXD015865.

Metabolomic data were analyzed using the KEGG compound database (MAVEN; Princeton NJ, USA).

For manuscripts utilizing custom algorithms or software that are central to the research but not yet described in published literature, software must be made available to editors/reviewers. We strongly encourage code deposition in a community repository (e.g. GitHub). See the Nature Research guidelines for submitting code & software for further information.

Data

Policy information about availability of data

All manuscripts must include a data availability statement. This statement should provide the following information, where applicable:

- Accession codes, unique identifiers, or web links for publicly available datasets
- A list of figures that have associated raw data
- A description of any restrictions on data availability

The mass spectrometry proteomics data have been deposited to the ProteomeXchange Consortium via the PRIDE partner repository with the dataset identifier PXD015865. Project Name: Label free quantitative (LFQ) proteomic analysis of mouse lymphatic endothelial cells and primary mouse dendritic cells in response to IFNgamma treatment in presence or absence of 3-HKA

Project accession: PXD015865

Username: reviewer34722@ebi.ac.uk

Password: seEaRVlC

Data are available to public upon receiving the DOI/pubmedID for the paper.

Processed raw files were saved in the PEAKS 8.5 projects for quantitative analysis based on MS1 area and further exported as "mzid" files for further processing and assessment of fold changes in the protein expression using MS2 exclusive spectra counts in the Scaffold suite software. The processed files are available on request and can be easily accessed and inspected using the viewer versions for each PEAKS (www.bioinfor.com/peaks-studio) and Scaffold software (www.proteomesoftware.com/products/scaffold).

List of figures containing information related to the metabolomic and proteomics raw files: Figure 1, Figure 2, Figure 4 Supplement Figure 1, Figure 2, Figure 4 Supplement Table S1, S2.

Metabolite assignments to KEGG compounds were performed using MAVEN (Princeton, NJ, USA) on the basis of accurate intact mass, high-resolution-based determination of chemical formulae, retention times against an in house standard library of ~1,000 compounds, including two custom synthesized isomers for this study for both 3-OH and 5-OH-kynureninamine. Peak areas were exported for comparative statistical analysis.

Field-specific reporting

Please select the one below that is the best fit for your research. If you are not sure, read the appropriate sections before making your selection.

☒ Life sciences ☐ Behavioural & social sciences ☐ Ecological, evolutionary & environmental sciences

For a reference copy of the document with all sections, see nature.com/documents/nr-reporting-summary-flat.pdf

Life sciences study design

All studies must disclose on these points even when the disclosure is negative.

Sample size

Data exclusions

Replication

Randomization

Blinding

Cytokines, WB analysis and metabolomic analysis were performed with a minimum of biological quintuplicate in each experiments. A total of overall 12-15 independent experiments were performed on human PBMC and mouse DCs. We followed the criteria for biological size samples as reported in https://www.ncbi.nlm.nih.gov/books/NBK43321/. We excluded two experiments (similar to the one depicted in Figure 4d-h) one because the IFN gamma stimulation did not work in the control sample (no up-regulation of STAT-1 and inflammatory cytokines was observed), the other because the base line (no stimulation) was already very high (STAT 1 activation); as such we could not compare it with the 3-HKA treatment. We also excluded 3 metabolomic experiments as the one depicted in Figure 1e, because the level of 3HKA was too low to detect in the untreated samples so it was not possible to calculate the fold increase upon cytokine stimulation.Cell treatment: validation of NFkb and STAT1 pathways by Western Blot: at least 10-12 independent cell treatment and at least five WB membranes replicates for probing with the corresponding antibodies. IDO presence in LEC was validated using PCR (3 biological replicates), immuno-staining (3 biological replicates) WB analysis (4 biological replicates) and MS/MS (2 biological replicates). Metabolomic experiments were repeated four times for detection of all tryptophan metabolites and 7 times for detection of 3-HKA. 3 metabolomic experiments were excluded because 3HKA levels in untreated samples were below the detection limit. Animal studies were performed in double blind between the post-doc and the pathologist. Cytokine analysis was performed on blinded samples submitted to Dr Gadina (NIH); similarly proteomic and metabolomic data were analyze in blind by the proteomic and metabolomic facilities (Columbia University and U. Colorado). The only experiments that were not performed in blind were the WB analysis and the immunohistochemistry. Animal treatment (Psoriasis Nephrotoxic Lupus) were not blind, however, all the histological analysis, cytokines quantitifaction and FACS analysis were in blind.
Allocation of cells and mice, for each treatment was random

Reporting for specific materials, systems and methods

We require information from authors about some types of materials, experimental systems and methods used in many studies. Here, indicate whether each material, system or method listed is relevant to your study. If you are not sure if a list item applies to your research, read the appropriate section before selecting a response.

Materials & experimental systems

n/a	Involved in the study
☐	☒ Antibodies
	X Eukaryotic cell lines
☒	☐ Palaeontology
☐	☒ Animals and other organisms
☒	☐ Human research participants
☒	☐ Clinical data

Methods

n/a	Involved in the study
☒	☐ ChIP-seq
☒	☐ Flow cytometry
☒	☐ MRI-based neuroimaging

Antibodies

Antibodies used

IDO1 clone 2E2 (dilution 1: 2000) (Enzo Lifescience Cat # ALX-804-902-0100); IDO2 (dilution 1:500) (Santa Cruz cat.# sc-374159); anti-human STAT1 (dilution 1:1000), which detects both STAT1 alpha (91 kDa) and STAT1 beta (84 kDa) isoforms, (CST; cat # 9172); anti-human Phospho-Stat1 (Tyr701) (dilution 1:1000) (CST; cat # 7649) (MW 91 and 84 kDa for phospho STAT-1); anti-human NF-κB p65 (dilution 1:800), (CST; cat # 8242), (MW 65 kDa for NF-kB p65); anti-human Phospho-NF-κB p65 (Ser536) (dilution 1:800), (CST; cat # 3031), (MW 65 kDa for Phospho-NF-κB p65); anti-human Phospho-IκBα (Ser32) (dilution 1:800) (CST; cat # 2859), (MW 40 kDa for Phospho-IκBα); anti-human Phospho-IκBα (Ser32/36) (dilution 1:1000) (CST; cat # 9246), (MW 40 kDa for Phospho-IκBα); anti-human IκBα (dilution 1:1000) (CST; cat # 4812), (MW 39kDa for IκBα); anti-human phospho-p44/42 MAPκ (Erk1/2) (Thr202/Tyr204) (197G2) (dilution 1:800) (CST; cat # 4377), (MW 42, 44 kDa for p-Erk1/2). Equal sample loading was determined using the goat polyclonal anti-beta actin antibody (dilution 1:1000) (Abcam, cat # ab8229) and GAPDH (Sigma cat # G9545, dilution 1:2000). Goat anti rat IgG (Thermo Fisher; Cat.# 31470, dilution 1:2000); Goat anti-rabbit IgG HRP conjugate (Southern Biotech; cat.# 4055-05, dilution 1:2000); Goat anti-rabbit IgG HRP-conjugated (R&D Systems; cat # HAF008, dilution 1:1000); Goat anti mouse (Southern Biotech; cat.# 1031-05, dilution 1:2000), Bovine anti-goat IgG-HRP (Santa Cruz Biotechnology, cat.# sc2350, dilution 1:1000). AF488-conjugated anti mouse Ido1 (Santa Cruz: cat # 53978, dilution 1:500) or (ENZO cat # ALX-804-902-0100, dilution 1:200); Lyve 1(Novus Biological cat # BAF2125, dilution 1:100); biotin conjugated anti mouse Podoplanin (Biolegend cat # 127403, dilution 1:200), overnight at 4oC.After washing thrice with 1X PBST, cells were incubated with the following secondary antibodies, Anti-Goat Alexa Fluor 405 (Abcam, cat #ab175664, dilution 1:100) and streptavidin AF 568 conjugate (molecular probes, Cat # S11226, dilution 1:200)

Validation

Animals and other organisms

Policy information about studies involving animals; ARRIVE guidelines recommended for reporting animal research

Laboratory animals	See reply
Wild animals	NA
Field-collected samples	NA
Ethics oversight	Experimental protocols used in this study were approved by Animal Care and Use Committee of Weill Cornell Medicine and by the Committee for Animal Experiments of the District of Southern Finland

Note that full information on the approval of the study protocol must also be provided in the manuscript.
